# Efficacy and Safety of Lianhua Qingke Tablets in Children With *Mycoplasma pneumoniae* Pneumonia: A Randomized, Double‐Blind, Multicenter, Placebo‐Controlled Clinical Trial

**DOI:** 10.1111/crj.70204

**Published:** 2026-06-25

**Authors:** He Wang, Haiwei Dou, Guangying Chen, Yanmin Wei, Hua Li, Shuqiong Xu, Rongzhen Kang, Shanhong Liu, Long Zhang, Nan Li, Hongli Sun, Chi Wang, Jie Zhao, Jirong Yi, Wentao Song, Hongrong Li, Rong Ma, Kunling Shen, Deli Xin, Wenjie Qi

**Affiliations:** ^1^ Beijing Friendship Hospital, Capital Medical University Beijing China; ^2^ Beijing Fengtai District Hospital of Traditional Chinese Medicine Beijing China; ^3^ Seventh, Hebei Province People's Hospital Hebei China; ^4^ Hebei Yiling Hospital Hebei China; ^5^ Mianyang Maternal and Child Health Hospital Sichuan China; ^6^ Jintang First People's Hospital Sichuan China; ^7^ Jinzhou People's Hospital Hebei China; ^8^ Qilu Hospital of Shandong University Dezhou Hospital Shandong China; ^9^ Xintai Second People's Hospital Shandong China; ^10^ Shijiazhuang Maternal and Child Health Care Hospital Hebei China; ^11^ Wen Deng District Integrated Traditional Chinese and Western Medicine Hospital of Weihai City Shandong China; ^12^ Guanghan People's Hospital Sichuan China; ^13^ Zibo Central Hospital Shandong China; ^14^ Mengyin County People's Hospital Shandong China; ^15^ Jiaozuo People's Hospital Henan China; ^16^ Hebei Luoxue Innovation Medical Research Institute State Key Laboratory for Innovation and Transformation of Luobing Theory Hebei China; ^17^ First Teaching Hospital of Tianjin University of Traditional Chinese Medicine National Clinical Research Center for Chinese Medicine Acupuncture and Moxibustion Tianjin China; ^18^ Beijing Children's Hospital, Capital Medical University Beijing China

**Keywords:** children, Lianhua Qingke tablets, macrolide resistance, *Mycoplasma pneumoniae* pneumonia, randomized double‐blind controlled trial

## Abstract

**Background:**

*Mycoplasma pneumoniae* (MP) is a major cause of community‐acquired pneumonia in children, with increasing macrolide resistance complicating treatment. Traditional Chinese Medicine (TCM) provides unique benefits for pediatric respiratory infections.

**Purpose:**

To evaluate the safety and efficacy of Lianhua Qingke (LHQK) tablets as adjunctive therapy in children with *M. pneumoniae* pneumonia (MPP).

**Study Design:**

This prospective, multicenter, randomized, double‐blind, placebo‐controlled trial adhered to the Declaration of Helsinki and was registered in the Chinese Clinical Trial Registry (ChiCTR2300078209).

**Methods:**

A total of 160 children aged 4–14 years with mild MPP were randomized 1:1 to receive conventional therapy plus LHQK or conventional therapy plus placebo for 7 days across 13 hospitals over 4 months. Efficacy analysis included 128 participants with centrally confirmed MP infection; safety analysis included all 160. Baseline characteristics were comparable (all *p* > 0.05), with 98.8% medication compliance in both groups (*p* > 0.05). The primary outcome was the major symptom resolution rate by Day 7.

**Results:**

The LHQK group showed a significantly higher major symptom resolution rate (85.9% vs. 65.6%, *p* < 0.05), with a numerically shorter median time to resolution (4.5 vs. 5.0 days, *p* = 0.072). LHQK also significantly improved cough and expectoration relief rates (all *p* < 0.05). No differences were found in laboratory parameters. Adverse event incidence was lower with LHQK (2.5% vs. 8.8%, *p* > 0.05), with no serious events reported.

**Conclusion:**

LHQK is a safe and effective adjunctive TCM therapy for mild MPP in children, offering significant benefits in respiratory symptom alleviation and a favorable safety profile, with a trend toward shorter recovery time, providing a valuable treatment option amid rising antibiotic resistance.

## Graphical abstract

In this randomized, double‐blind, placebo‐controlled trial involving 160 children with mild *Mycoplasma pneumoniae* pneumonia, adding Lianhua Qingke tablets to conventional therapy significantly improved symptom resolution by Day 7 (85.9% vs. 65.6%) and shortened median recovery time (4.5 vs. 5.0 days), particularly for cough and sputum, with fewer adverse events (2.5% vs. 8.8%) and no severe reactions.

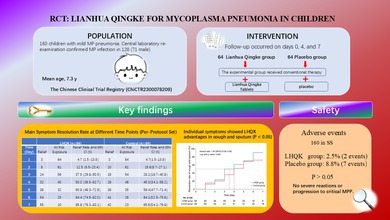

**Abbreviations**
CI: Confidence interval—A range of values (e.g., 95% CI) that likely contains the true population parameter, used to estimate the precision of statistical results
CRP: C‐reactive protein—A biomarker of inflammation measured in blood, elevated in infections like pneumonia
LHQK: Lianhua Qingke—A Traditional Chinese Medicine tablet formulation used as adjunctive therapy for respiratory conditions like *M. pneumoniae* pneumonia
MP:
*M. pneumoniae* —An atypical bacterium that causes respiratory infections, particularly pneumonia in children
MPP:
*M. pneumoniae* pneumonia—Pneumonia caused by *M. pneumoniae* , often presenting with cough and fever
SD: Standard deviation—A measure of variability in data, indicating how much values deviate from the mean
TCM: Traditional Chinese Medicine—A holistic medical system originating from China using herbs, acupuncture, and theories like Qi and meridians to treat imbalances
WBC: White blood cell—Immune cells in the blood that fight infections; counts are monitored for signs of infection
χ^2^
: Chi‐square test—A statistical test used to compare observed and expected frequencies in categorical data, assessing associations between variables

## 1 Introduction

*M. pneumoniae* (MP) is a major cause of community‐acquired pneumonia in children, accounting for 10% to 40% of cases [1]. Although most MPP cases are self‐limiting, they can lead to severe pulmonary infections and extrapulmonary complications, threatening pediatric health [2]. Macrolide antibiotics are the first‐line treatment in China, but rising resistance has reduced their efficacy, prolonging fever and symptoms [3].

Traditional Chinese Medicine (TCM) offers a complementary approach for pediatric respiratory infections [4]. TCM theory attributes MPP to children's physiological immaturity, making them susceptible to pathogens that invade via the mouth and nose, generating internal heat, scorching lung fluids, and forming phlegm. This manifests as patterns like wind‐heat attacking the exterior or phlegm‐heat obstructing the lung [5, 6]. Lianhua Qingke (LHQK) tablets address these patterns by diffusing the lung, discharging heat, transforming phlegm, and relieving cough to clear the airway obstruction. Derived from classical prescriptions like Ma Xing Shi Gan Tang and Qing Jin Hua Tan Tang, LHQK comprises herbs such as Honeysuckle (*Lonicerae Japonicae* Flos, Jinyinhua), *Forsythia suspensa* (Forsythiae Fructus, Lianqiao), and Burdock seed (Arctii Fructus, Niubangzi), which form an acrid‐cool cohort that releases the exterior and expels heat. Mulberry root bark (Mori Cortex, Sangbaipi), *Scutellaria baicalensis* (Scutellariae Radix, Huangqin), and Gypsum (Gypsum Fibrosum, Shigao) work together to clear lung heat, an action reinforced by Rhubarb (Rhei Radix et Rhizoma, Dahuang), which drains heat via the bowels—a method of “purging the lung through purgation.” Simultaneously, Pinellia (Pinelliae Rhizoma, Banxia), *Zhejiang fritillaria* (Fritillariae Zhebeimu, Zhebeimu), Peucedanum (Peucedani Radix, Qianhu), and tangerine peel (Citri Reticulatae Pericarpium, Chenpi) act in concert to resolve phlegm and rectify Qi dynamics. Finally, Ephedra (Ephedrae Herba, Mahuang), bitter almond (Armeniacae Semen Amarum, Kuxingren), and Platycodonis Radix (Platycodonis Radix, Jiegeng) serve to diffuse and descend lung Qi, restoring its normal functions of diffusion and descent, and targeting exterior release, heat clearance, phlegm resolution, and lung Qi restoration [7]. This multi‐targeted formula promotes lung Qi flow, phlegm elimination, and cough cessation.

Despite clinical potential, high‐quality evidence for LHQK in MP infections is limited. This randomized, double‐blind, multicenter, placebo‐controlled trial evaluated LHQK's efficacy and safety as adjunctive therapy in children with MPP.

## 2 Materials and Methods

### 2.1 Study Design

This prospective, multicenter, randomized, double‐blind, placebo‐controlled clinical trial evaluated the safety and efficacy of LHQK tablets as adjunctive therapy for MPP in children. The protocol adhered to the Declaration of Helsinki, with ethics approval from each participating center's committee. The trial was registered in the Chinese Clinical Trial Registry (ChiCTR2300078209) on November 30, 2023, before the enrollment of the first patient on December 9, 2023.

### 2.2 Treatment Protocol

All patients received conventional therapy according to the 2023 Chinese guidelines for pediatric MPP [1], standardized across all 13 centers. Antibiotic therapy consisted of macrolides as first‐line treatment: azithromycin (10 mg/kg once daily for 3 days, maximum 500 mg per day), administered orally or intravenously. Alternative macrolides or second‐line antibiotics (e.g., doxycycline for children ≥ 8 years) were permitted as needed. Supportive care included antipyretics (ibuprofen or acetaminophen) for fever > 38.5°C, antitussive/expectorant medications (e.g., ambroxol, acetylcysteine) as needed, and intravenous fluids when clinically indicated. Systemic corticosteroids (methylprednisolone) were reserved for patients with severe inflammation; none of the patients in this trial received corticosteroids. The experimental group received conventional therapy plus age‐adjusted LHQK tablets (two tablets for children aged < 7 years, four tablets for children aged ≥ 7 years), administered three times daily. The control group received conventional therapy plus a placebo (identical in appearance, weight, smell, and taste, but inactive). Follow‐up was conducted on days 0, 4, and 7. A 7‐day treatment duration was selected because it aligns with the typical course of macrolide therapy (3–5 days) while allowing adequate time to detect the adjunctive effects of LHQK. This duration is also consistent with previous LHQK trials in acute bronchitis and COVID‐19, and it covers the expected natural course of mild MPP symptoms.

**FIGURE 1:**
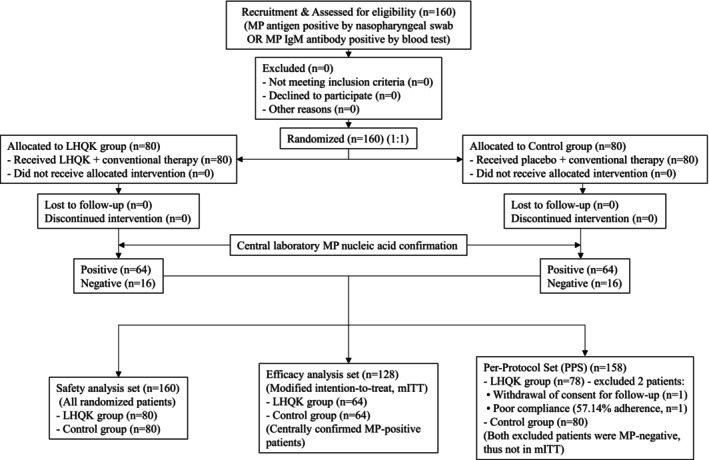
A total of 160 children who tested positive for MP by either nasopharyngeal swab antigen test or blood IgM antibody test were assessed for eligibility. All 160 met the inclusion criteria and were enrolled. No patients were excluded prior to randomization. Patients were randomly assigned in a 1:1 ratio to receive either LHQK plus conventional therapy (*n* = 80) or placebo plus conventional therapy (*n* = 80). All 160 patients completed the 7‐day treatment and follow‐up period; there were no losses to follow‐up, discontinuations of intervention, or protocol violations. After randomization, central laboratory MP nucleic acid testing was performed for all patients to confirm the diagnosis. A total of 128 patients (64 in the LHQK group and 64 in the control group) tested positive for MP and were included in the mITT efficacy analysis. The remaining 32 patients (16 in each group) tested negative and were therefore excluded from the efficacy analysis; however, all 160 patients (including the 32 negative cases) were retained in the safety analysis. Two additional patients in the LHQK group were excluded from the PPS due to withdrawal of consent for follow‐up examinations (*n* = 1) and poor compliance (57.14% adherence, *n* = 1); both were MP‐negative and thus not part of the mITT population. No other exclusions were applied. Abbreviations: IgM, immunoglobulin M; LHQK, Lianhua Qingke; mITT, modified intention‐to‐treat; MP, *Mycoplasma pneumoniae* ; PPS, per‐protocol set.

Randomization was performed in a 1:1 ratio using a computer‐generated sequence (SAS 9.4; SAS Institute, Cary, NC, USA) with a block size of four, prepared by an independent statistician. Allocation concealment was ensured using sequentially numbered, sealed, opaque envelopes. After enrollment, the pharmacist opened the envelope and dispensed the assigned medication. Investigators, outcome assessors, participants, and data analysts were all blinded to treatment allocation throughout the study. Medication was assigned sequentially by enrollment number, with blinding maintained throughout the 7‐day period. Compliance was assessed by returned medication counts (acceptable: 80%–120% adherence; compared via χ2 test or Fisher's exact test). Unblinding was restricted to emergencies; rates exceeding 20% would invalidate the design. Any early unblinding or disclosure would result in withdrawal (Figure 1).

### 2.3 Macrolide Resistance Testing

Macrolide resistance testing was performed on nasopharyngeal swab samples from enrolled patients. The 23S rRNA gene was amplified by polymerase chain reaction, and the amplicons were sequenced to screen for known macrolide resistance–associated mutations, including A2063G, A2064G, and A2067G. The presence of any of these mutations was recorded as positive for macrolide resistance. Subgroup analyses based on resistance status were not prespecified due to sample size limitations.

### 2.4 Patients

#### 2.4.1 Inclusion Criteria

Inclusion criteria included age 4–14 years, meeting diagnostic criteria for mild MPP, positive MP antigen via nasopharyngeal swab or immunoglobulin M antibodies in blood, illness duration of 24 h to 5 days with axillary temperature ≥ 37.3°C, TCM diagnosis of wind‐heat invading the lungs, and voluntary guardian consent (with child assent if ≥ 8 years). Confirmed cases (positive central lab re‐examination for MP antibodies/nucleic acids) were included in efficacy analysis; patients with negative results were excluded from microbiological diagnosis but included in safety analysis.

#### 2.4.2 Exclusion Criteria

Exclusion criteria included refractory/severe MPP, significant lung tumors/tuberculosis on imaging, acute infections (e.g., measles, pertussis), asthma, foreign bodies, malnutrition, immunodeficiency, severe comorbidities, recent drug trials, allergies to LHQK components, or other reasons deemed inappropriate by the investigators.

#### 2.4.3 Rejection and Shedding Criteria

Withdrawal criteria included allergic reactions, serious adverse events; other conditions that affected the assessment of efficacy and safety during the trial; poor compliance (defined as taking < 80% or > 120% of the experimental medication); changes in medications, adding prohibited drugs, or other protocol violations; blinding interruption; condition worsening, with subsequent treatment discontinuation (these children were withdrawn from the trial as ineffective cases after completing the necessary laboratory tests, with effective treatment measures implemented); and serious violations of inclusion or exclusion criteria discovered after randomization.

Self‐initiated withdrawal occurred if the patient withdrew from the clinical research for any reason (informing the attending doctor to discontinue participation) or if the subject did not explicitly request to withdraw but stopped taking medication and attending follow‐up visits (with the research doctor attempting to understand the reasons for withdrawal).

Efforts were made to complete follow‐up for withdrawn participants, with reasons documented. All cases of withdrawal were documented in the trial summary table, along with the reasons for withdrawal in the case report form.

### 2.5 Outcome Measures

#### 2.5.1 Efficacy Outcome Measures

Efficacy outcomes were analyzed only for children with confirmed MP infection via central laboratory re‐examination of antibodies and/or nucleic acids to ensure reliability. All enrolled children were included in the safety analysis.

##### 2.5.1.1 Primary Efficacy Endpoint

The primary endpoint was the major symptom resolution rate: the proportion of patients with at least a 1‐point reduction in each major symptom (fever, cough, sputum production, headache, sore throat) by Day 7, sustained for ≥ 24 h.

##### 2.5.1.2 Secondary Efficacy Endpoints

Secondary endpoints included: TCM syndrome effective rate (≥ 50% score decrease); resolution rates and times for individual symptoms (fever, cough, sputum, headache, sore throat); complete symptom disappearance rates and times; proportions of patients progressing to severe or critical MPP; and changes in C‐reactive protein (CRP) levels from baseline to Day 7. All secondary endpoints were considered exploratory. No multiplicity adjustment was applied for secondary analyses; therefore, findings for secondary endpoints should be interpreted as hypothesis‐generating and require confirmation in future studies.

#### 2.5.2 Symptom Assessment

All symptom assessments were performed using a standardized, pre‐defined symptom scoring scale (0 = no symptom, 1 = mild, 2 = moderate, 3 = severe for each symptom). The scoring criteria were adapted from the TCM syndrome scoring system and defined in the study protocol before trial initiation. Assessments were conducted by trained pediatricians at each participating center, not by parents or guardians, to ensure professional and consistent evaluation. Inter‐center consistency was maintained through: (i) a centralized training session for all investigators prior to study start, during which the scoring criteria were reviewed with case examples; (ii) a detailed instruction manual provided to each center; (iii) regular monitoring visits by the coordinating center to review source documents and ensure adherence; and (iv) central review of case report forms for data completeness and consistency. The reproducibility and objectivity of symptom assessments are supported by the high inter‐rater agreement observed during monitoring visits.

#### 2.5.3 Safety Outcome Measures

Safety outcome measures included the incidence of adverse events/adverse drug reactions, assessed at the conclusion of the study period, and vital signs (e.g., blood pressure, heart rate, respiratory rate, temperature) and laboratory tests (including complete blood count, urinalysis, and liver and kidney function tests such as alanine aminotransferase, creatinine, and bilirubin), assessed at baseline and at the end of treatment (selected based on clinical practice and patient needs). All adverse events were graded for severity (mild, moderate, severe) according to common terminology criteria. The relationship between adverse events and study medication was assessed by the investigators as unrelated, unlikely related, possibly related, probably related, or definitely related. Laboratory abnormalities were classified as clinically significant or not clinically significant based on predefined thresholds.

### 2.6 Sample Size

The sample size was calculated based on the primary endpoint of major symptom resolution rate on Day 7. Assuming a 75.0% resolution rate in the LHQK group and 50.0% in the placebo group, with a two‐sided alpha of 0.05, 80% power, and a 1:1 allocation ratio, statistical software (PASS 21.0, NCSS, LLC, Kaysville, Utah, USA) estimated a total sample size of 110 participants. Accounting for a 14.6% false‐positive rate in initial MP diagnostic tests and a 20.0% exclusion rate due to negative central laboratory re‐examination for MP antibodies or nucleic acids, the adjusted sample size was 158 participants. Thus, 160 participants were enrolled to ensure adequate power, providing a conservative estimate for detecting clinically meaningful benefits of LHQK as an adjunct to conventional therapy.

### 2.7 Informed Consent Process

Researchers explained study procedures, risks, benefits, and alternatives to children and guardians, allowing time for questions. Guardians (and children ≥ 8 years) signed consent forms; a witness assisted illiterates. Child autonomy was respected if refusal occurred despite guardian consent. Withdrawals were honored immediately.

### 2.8 Multicenter Study Information

This multicenter trial enrolled 160 patients from December 9, 2023, to March 29, 2024, across 13 hospitals: Hebei Yiling Hospital, Jinzhou People's Hospital, Mengyin County People's Hospital, Xintai Second People's Hospital, the Second Affiliated Hospital of Hebei University of Traditional Chinese Medicine, Weihai Wendeng Integrated Traditional Chinese and Western Medicine Hospital, Qilu Hospital of Shandong University Dezhou Hospital, Mianyang Maternal and Child Health Hospital, Jiaozuo People's Hospital, Guanghan People's Hospital, Shijiazhuang Maternal and Child Health Hospital, Jintang County First People's Hospital, and Zibo Central Hospital. The trial was registered in the Chinese Clinical Trial Registry (ChiCTR2300078209).

### 2.9 Statistical Analysis

All statistical tests were two‐sided, with a significance level of *p* < 0.05. Measurement data are presented as mean ± standard deviation (or range; non‐normally distributed data as median and quartiles). Count data are reported as numbers or proportions. Quantitative data comparisons used *t*‐tests or Wilcoxon rank‐sum tests based on distribution. Time‐to‐event data are described as median with 95% confidence intervals (CIs). Categorical data were compared using chi‐square or exact probability tests, and ordinal data using Wilcoxon rank‐sum or Cochran–Mantel–Haenszel tests. Symptom resolution rates were estimated via Kaplan–Meier analysis, with log‐rank tests for inter‐group comparisons. Adverse event incidence was compared using chi‐square or Fisher's exact tests. Missing data were handled via available case analysis without imputation. Analyses were conducted using SAS version 9.4 (SAS Institute). Given the exploratory nature of the secondary endpoints, no adjustment for multiple comparisons was performed. The primary endpoint was tested at a two‐sided significance level of α = 0.05. Secondary analyses were conducted without correction for multiplicity; thus, results for secondary endpoints should be interpreted with caution as hypothesis‐generating.

The normality of continuous variables was assessed using the Shapiro–Wilk test. Normally distributed data were analyzed using independent *t*‐tests, while non‐normally distributed data were analyzed using Wilcoxon rank‐sum tests. Missing data were handled using available case analysis; no imputation was performed for missing values. Effect sizes were reported as rate differences with 95% CIs for categorical outcomes and as hazard ratios with 95% CIs for time‐to‐event outcomes. All statistical tests were two‐sided, and a *p*‐value < 0.05 was considered statistically significant for the primary endpoint. Given the exploratory nature of the secondary endpoints, no adjustment for multiple comparisons was applied, and findings for secondary endpoints should be interpreted as hypothesis‐generating.

#### 2.9.1 Analysis Populations

The safety analysis set included all randomized patients who received at least one dose of the study medication (*n* = 160). The full analysis set (FAS) included all randomized patients (*n* = 160) and was used for baseline comparisons and secondary analyses. The primary efficacy analysis was performed on the modified intention‐to‐treat (mITT) population, which consisted of all randomized patients with centrally confirmed MP infection (positive nucleic acid test). This population included 128 patients (64 per group) and was prespecified in the statistical analysis plan to ensure diagnostic accuracy, given the known false‐positive rate (14.6%) of rapid MP tests. A per‐protocol set (PPS) was also defined, excluding patients with major protocol violations. The PPS included 158 patients (78 in the LHQK group and 80 in the control group); the two excluded patients (both in the LHQK group) were excluded due to withdrawal of consent for follow‐up examinations (*n* = 1) and poor compliance (57.1% adherence, *n* = 1). Neither of these patients was MP‐positive, and therefore they were not part of the mITT population. All randomized patients were included in the safety analysis.

#### 2.9.2 Sensitivity Analysis

To assess whether the exclusion of MP‐negative patients from the efficacy analysis introduced selection bias, we compared baseline demographic and clinical characteristics between the included (MPP, *n* = 128) and excluded (non‐MPP, *n* = 32) patients using data from the FAS. No significant differences were observed in age, sex, disease duration, baseline CRP levels, or TCM syndrome scores (all *p* > 0.05; see Table S1). This suggests that the exclusion of MP‐negative patients did not materially affect the comparability of the two groups.

## 3 Results

### 3.1 Patient Characteristics

A total of 160 children with mild MP pneumonia were enrolled and randomized (80 per group). Central laboratory MP nucleic acid testing confirmed MP infection in 128 patients (64 per group), who constituted the mITT population for the primary efficacy analysis. The remaining 32 patients (16 per group, 20.0% of the randomized population) tested negative and were therefore excluded from the efficacy analysis but were retained in the safety analysis. No patients were lost to follow‐up or discontinued treatment. Two patients in the LHQK group were excluded from the per‐protocol analysis due to withdrawal of consent for follow‐up examinations (*n* = 1) and poor compliance (57.1% adherence, *n* = 1); both were MP‐negative and thus not part of the mITT population. Baseline characteristics were well‐balanced between the LHQK and control groups in the mITT population (*p* > 0.05 for all variables; Table 1). Medication adherence was high and comparable (98.8% in both groups).

**TABLE 1:** Baseline clinical characteristics of patients in the per‐protocol analysis set.

Variable	LHQK (*n* = 64)	Control (*n* = 64)	*p*
Age	7.6 ± 2.5	7.1 ± 2.1	0.189
< 7 years old *n* (%)	24 (37.5)	28 (43.8)	0.472
Male *n* (%)	33 (51.6)	38 (59.4)	0.374
Weight (kg)	30.28 ± 13.08	28.91 ± 12.46	0.545
BMI (kg/m^2^)	16.87 ± 3.29	16.92 ± 3.34	0.930
Body temperature (°C)	37.34 ± 0.91	37.36 ± 0.81	0.910
Illness duration (hours)	89.0 (69.5–100.0)	90.5 (71.5–102.0)	0.744
Pulse (times/min)	96.89 ± 10.54	95.91 ± 10.04	0.589
Breath (times/min)	23.41 ± 2.74	23.50 ± 2.56	0.842
White blood cell count (× 10^9^/L)	8.11 ± 3.41	8.08 ± 3.53	0.964
Percentage of neutrophils (%)	59.97 ± 11.90	60.98 ± 11.47	0.629
CRP (mg/L)	12.39 ± 11.99	11.41 ± 15.03	0.692
Alanine aminotransferase (μ/L)	18.91 ± 22.49	16.99 ± 15.18	0.543
Total bilirubin (μmol/L)	7.27 ± 3.15	7.68 ± 3.31	0.441
Creatinine (μmol/L)	38.02 ± 9.24	38.30 ± 7.63	0.847

*Note:* Data are presented as mean ± standard deviation for continuous variables or as number (percentage) for categorical variables. Between‐group comparisons at baseline were performed using the independent *t*‐test for continuous variables and the χ^2^ test for categorical variables. Since the illness duration data were not normally distributed, medians (interquartile ranges) are presented in the table, and between‐group comparisons were performed using the Wilcoxon rank‐sum test. A *p*‐value > 0.05 indicates no significant difference between groups.

Abbreviations: BMI, body mass index; CRP, C‐reactive protein; LHQK, Lianhua Qingke.

### 3.2 Macrolide Resistance

Among the 128 patients with centrally confirmed MP infection, macrolide resistance testing was performed in 101 patients (78.9%). Sequencing of the 23S rRNA gene revealed the A2063G mutation in 38 patients (37.6%, 95% CI: 28.5%–47.4%), while no other known resistance mutations (e.g., A2064G, A2067G) were detected. The remaining 63 patients (62.4%) showed no resistance‐associated mutations. The A2063G mutation was detected in 20 patients in the LHQK group and 18 patients in the control group. These findings confirm a substantial prevalence of macrolide resistance in our cohort, predominantly mediated by the A2063G mutation. A subgroup analysis based on resistance status was not performed due to limited statistical power.

### 3.3 Primary Endpoints

**FIGURE 2:**
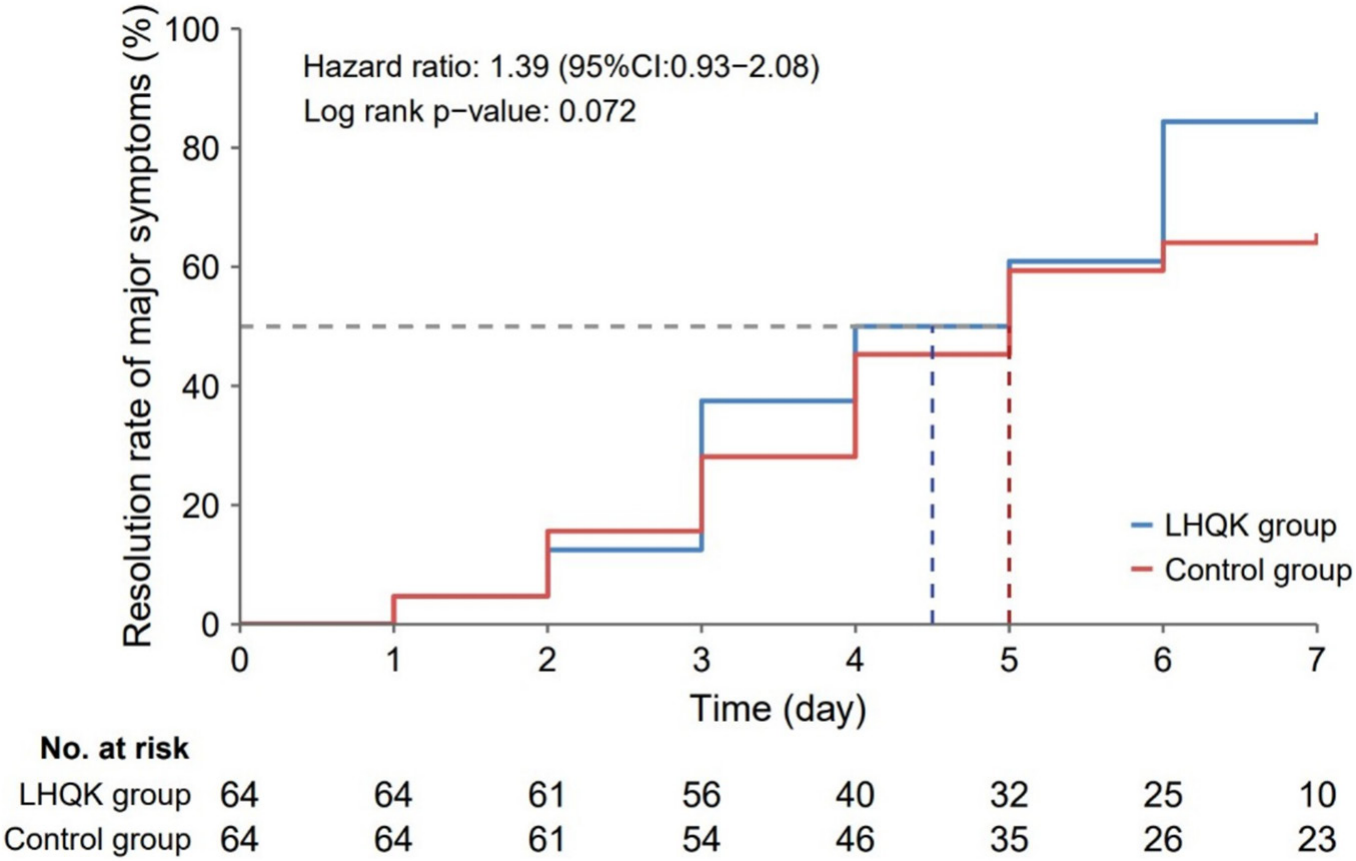
Kaplan–Meier curves for time to resolution of major symptoms (per‐protocol set). The primary outcome was time to resolution of a composite of major symptoms (fever, cough, sputum production, headache, and sore throat), defined as a reduction of at least one level in each symptom sustained for at least 24 h. Blue line: LHQK group; red line: control group. Groups were compared using the log‐rank test. Median time to resolution was 4.5 days in the LHQK group and 5.0 days in the control group (*p* = 0.072). The number of patients at risk at each time point is shown below the x‐axis. Abbreviation: LHQK, Lianhua Qingke.

**FIGURE 3:**
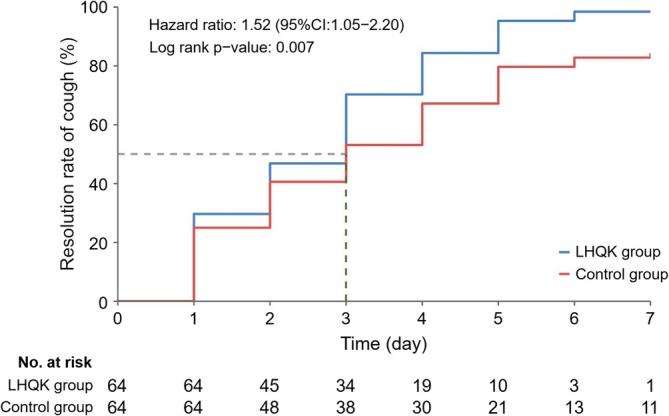
Kaplan–Meier curves for time to resolution of cough (per‐protocol set). Resolution was defined as a reduction of at least one level in cough severity sustained for at least 24 h. Blue line: LHQK group; red line: control group. Groups were compared using the log‐rank test. The LHQK group showed a significantly higher rate of cough resolution compared to the control group (*p* < 0.05). The number of patients at risk at each time point is shown below the x‐axis. Abbreviation: LHQK, Lianhua Qingke.

**FIGURE 4:**
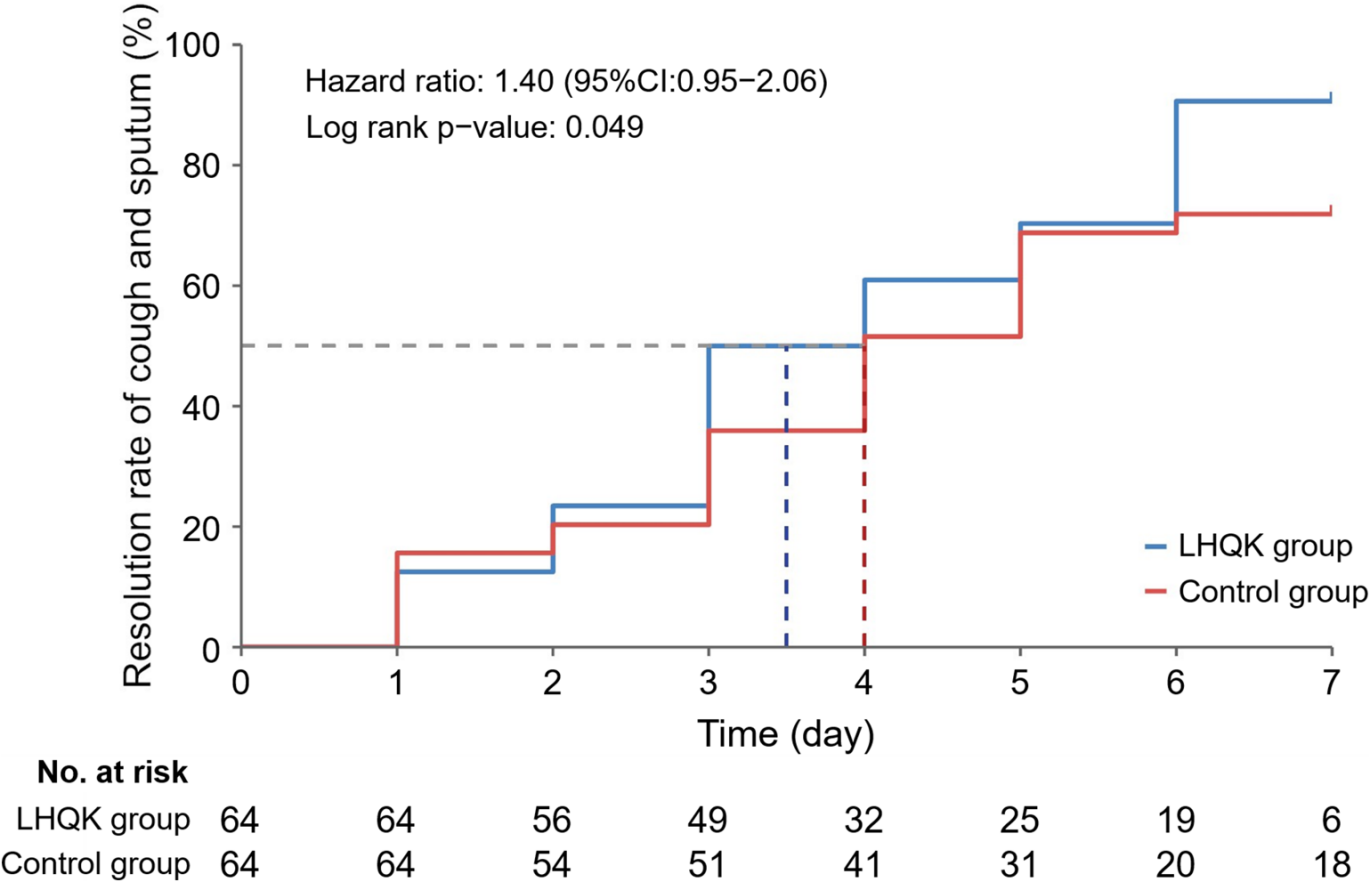
Kaplan–Meier curves for time to resolution of sputum production (per‐protocol set). Resolution was defined as a reduction of at least one level in sputum production severity sustained for at least 24 h. Blue line: LHQK group; red line: control group. Groups were compared using the log‐rank test. The LHQK group showed a significantly higher rate of sputum production resolution compared to the control group (*p* < 0.05). The number of patients at risk at each time point is shown below the x‐axis. Abbreviation: LHQK, Lianhua Qingke.

Symptom resolution rates were similar in the first 3 days but higher in the LHQK group from Day 4 onward (Table 2). By Day 7, rates were 85.9% versus 65.6% (*p* < 0.05; Table 3), with median resolution times of 4.5 versus 5.0 days (HR: 1.39, 95% CI: 0.93–2.08; *p* = 0.072; Figure 2). Individual symptoms showed LHQK advantages in cough and sputum (*p* < 0.05), with comparable fever and non‐significant sore throat benefits (*p* = 0.466; Table 4, Figures 3 and 4).

**TABLE 2:** Main symptom resolution rate at different time points (per‐protocol set).

Time (Day)	LHQK (*n* = 64)			Control (*n* = 64)		
	Relief	At‐risk exposure	Relief rate and 95% CI (%)	Relief	At‐risk exposure	Relief rate and 95% CI (%)
1	3	64	4.7 (1.5–13.8)	3	64	4.7 (1.5–13.8)
2	8	61	12.5 (6.5–23.4)	10	61	15.6 (8.7–27.1)
3	24	56	37.5 (26.9–50.5)	18	54	28.1 (18.7–40.9)
4	32	40	50.0 (38.5–62.7)	29	46	45.3 (34.1–58.2)
5	39	32	60.9 (49.3–72.8)	38	35	59.4 (47.7–71.4)
6	54	25	84.4 (74.5–92.0)	41	26	64.1 (52.5–75.6)
7	55	10	85.9 (76.3–93.1)	42	23	65.6 (54.1–76.9)

*Note:* The number of patients achieving symptom resolution at each time point is shown, with cumulative relief rates and 95% CIs calculated using the Kaplan–Meier method. The primary comparison of time to symptom resolution between groups was performed using the log‐rank test (see Figure 2).

Abbreviations: CI, confidence interval; LHQK, Lianhua Qingke.

**TABLE 3:** Analysis of main symptom resolution rate at Day 7 (per‐protocol set).

	LHQK	Control	Statistic	*p*
N (Nmiss)	64	64	7.194	0.007
Relieved, *n* (%)	55 (85.9)	42 (65.6)		
Not Relieved, *n* (%)	9 (14.1)	22 (34.4)		

*Note:* Data are presented as number (percentage). Between‐group comparison of the proportion of patients with symptom resolution by Day 7 was performed using the χ^2^ test.

Abbreviations: LHQK, Lianhua Qingke; Nmiss, number of missing values.

**TABLE 4:** Analysis of individual symptom resolution rates at Day 7 (per‐protocol set).

	LHQK	Control	Statistic	*p*	Method
Cough					
N (Nmiss)	64 (0)	64 (0)	8.056	0.005	Chisq
Relieved, *n* (%)	63 (98.4)	54 (84.4)			
Not relieved, *n* (%)	1 (1.6)	10 (15.6)			
Fever					
N (Nmiss)	59 (5)	59 (5)	NA	NA	NA
Relieved *n* (%)	59 (100.0)	59 (100.0)			
Not relieved, *n* (%)	0 (0.0)	0 (0.0)			
Sputum production					
N (Nmiss)	64 (0)	64 (0)	7.904	0.005	Chisq
Relieved, *n* (%)	59 (92.2)	47 (73.4)			
Not relieved, *n* (%)	5 (7.8)	17 (26.6)			
Throat redness and pain					
N (Nmiss)	52 (12)	45 (19)	NA	0.466	Fisher
Relieved, *n* (%)	49 (94.2)	40 (88.9)			
Not relieved, *n* (%)	3 (5.8)	5 (11.1)			

*Note:* Data are presented as number (percentage). Between‐group comparisons for each symptom were performed using the χ^2^ test or Fisher's exact test, as appropriate. NA: not applicable, as all patients with fever at baseline achieved relief.

Abbreviations: Chisq, chi‐square test; LHQK, Lianhua Qingke; NA, not applicable; Nmiss, number of missing values.

### 3.4 Secondary Endpoints

**FIGURE 5:**
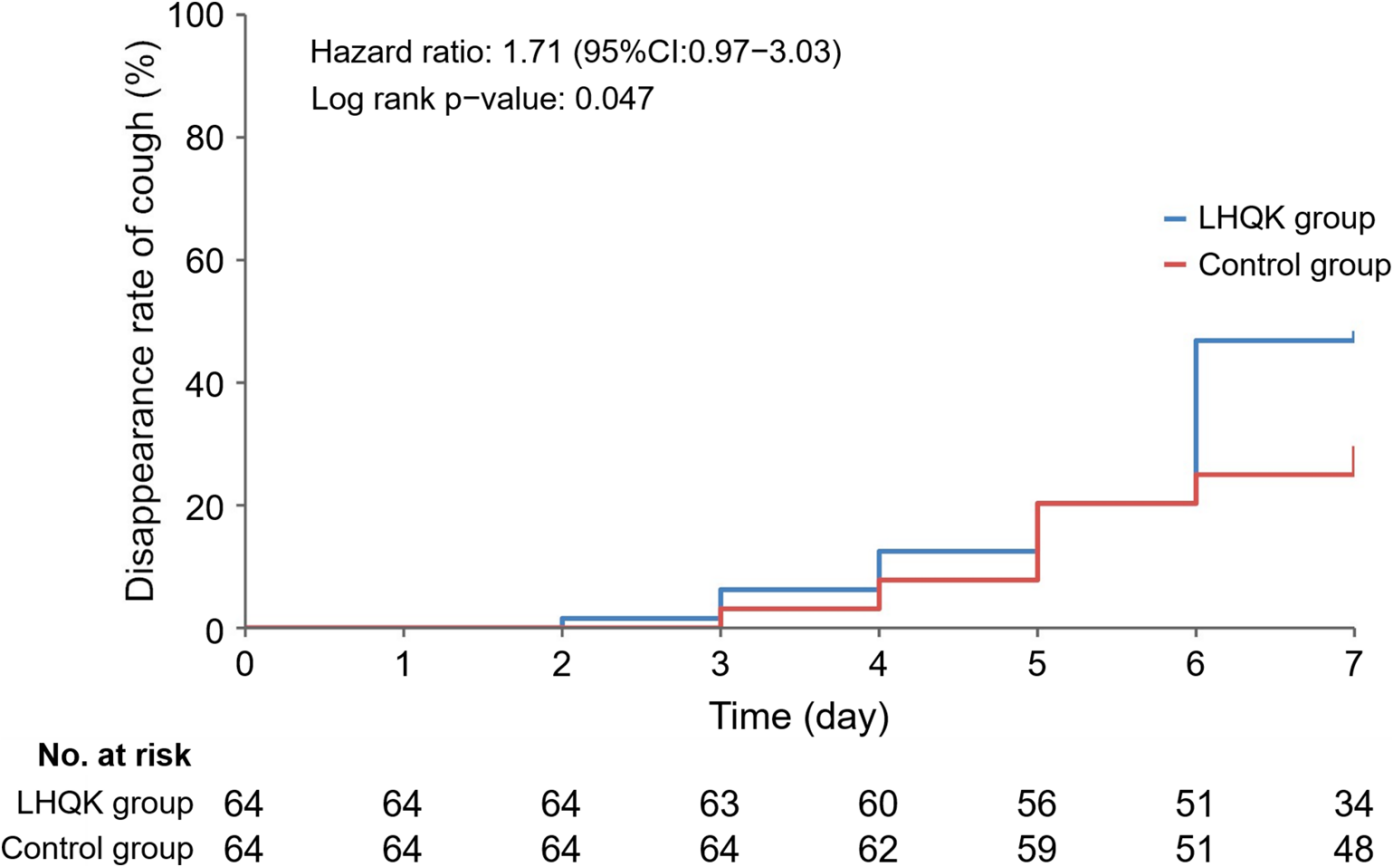
Kaplan–Meier curves for time to disappearance of cough (per‐protocol set). Disappearance was defined as the complete absence of the cough symptom. Blue line: LHQK group; red line: control group. Groups were compared using the log‐rank test. A significantly higher proportion of children in the LHQK group experienced cough disappearance by Day 7 compared to the control group (*p* < 0.05). The number of patients at risk at each time point is shown below the x‐axis. Abbreviation: LHQK, Lianhua Qingke.

**FIGURE 6:**
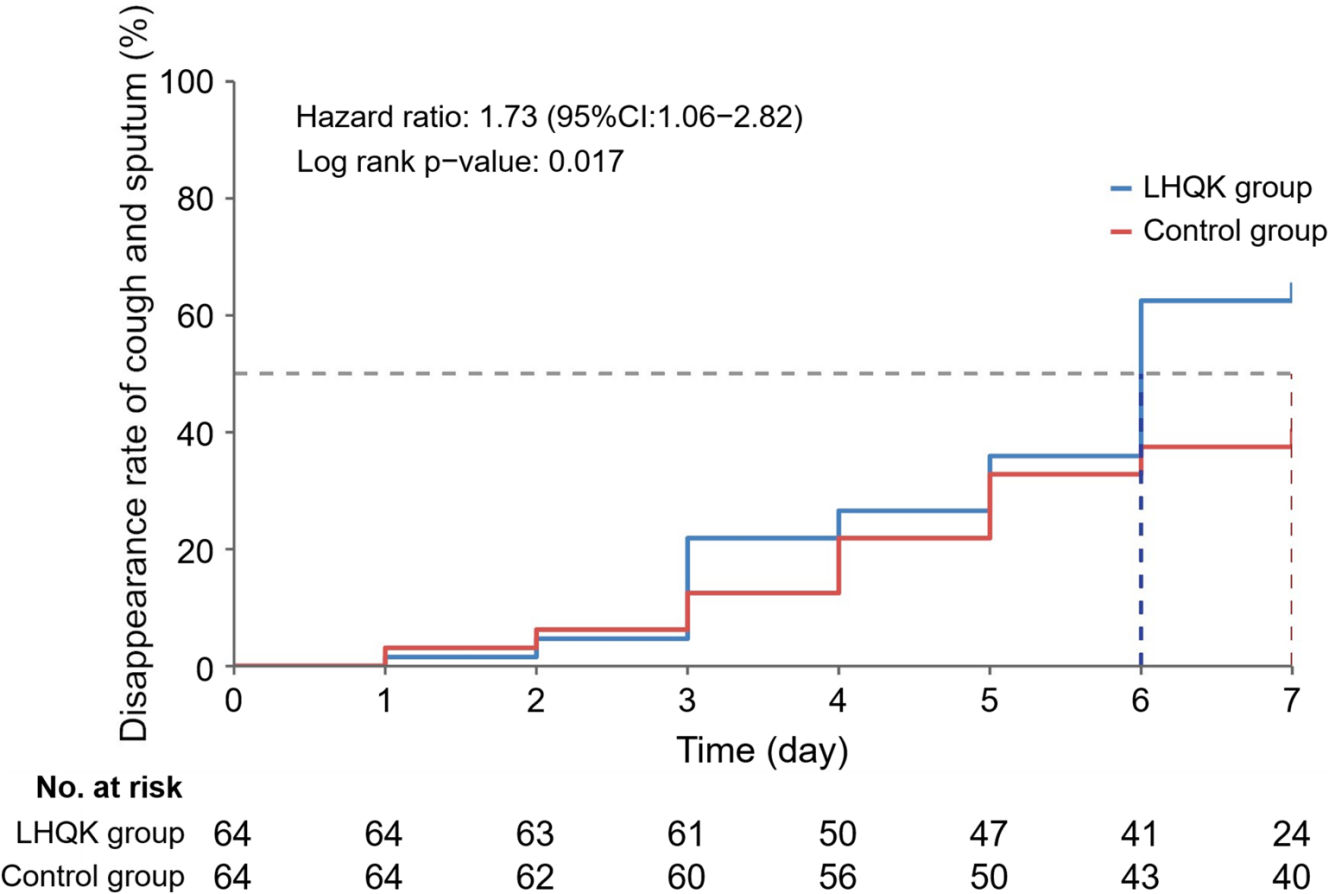
Kaplan–Meier curves for time to disappearance of sputum production (per‐protocol set). Disappearance was defined as the complete absence of the sputum production symptom. Blue line: LHQK group; red line: control group. Groups were compared using the log‐rank test. A significantly higher proportion of children in the LHQK group experienced sputum production disappearance by Day 7 compared to the control group (*p* < 0.05). The number of patients at risk at each time point is shown below the x‐axis. Abbreviation: LHQK, Lianhua Qingke.

Symptom disappearance rates were higher in the LHQK group for cough, sputum, and throat pain (*p* < 0.05; Table 5, Figures 5 and 6), with similar fever rates. TCM syndrome efficacy and significant efficacy rates improved more in the LHQK group (*p* < 0.05; Table 6). Effect sizes with 95% CIs are reported for all primary and secondary outcomes, and time‐to‐event analyses were performed using Kaplan–Meier methods with log‐rank tests.

**TABLE 5:** Analysis of individual symptom disappearance rates at Day 7 (per‐protocol set).

	LHQK	Control	Statistic	*p*	Method
Cough					
N (Nmiss)	64 (0)	64 (0)	4.726	0.030	Chisq
Disappeared, *n* (%)	31 (48.4)	19 (29.7)			
Did not disappear, *n* (%)	33 (51.6)	45 (70.3)			
Fever					
N (Nmiss)	59 (5)	59 (5)	NA	1.000	Fisher
Disappeared, *n* (%)	56 (94.9)	57 (96.6)			
Did not disappear, *n* (%)	3 (5.1)	2 (3.4)			
Sputum production					
N (Nmiss)	64 (0)	64 (0)	8.031	0.005	Chisq
Disappeared, *n* (%)	42 (65.6)	26 (40.6)			
Did not disappear, *n* (%)	22 (34.4)	38 (59.4)			
Headache					
N (Nmiss)	25 (39)	23 (41)	NA	1.000	Fisher
Disappeared, *n* (%)	22 (88.0)	21 (91.3)			
Did not disappear, *n* (%)	3 (12.0)	2 (8.7)			
Throat redness and pain					
N (Nmiss)	52 (12)	45 (19)	6.305	0.012	Chisq
Disappeared, *n* (%)	48 (92.3)	33 (73.3)			
Did not disappear, *n* (%)	4 (7.7)	12 (26.7)			

*Note:* Data are presented as number (percentage). Symptom disappearance was defined as the complete absence of the symptom. Between‐group comparisons for each symptom were performed using the χ^2^ test or Fisher's exact test, as appropriate.

Abbreviations: Chisq, chi‐square test; LHQK, Lianhua Qingke; NA, not applicable; Nmiss, number of missing values.

**TABLE 6:** Analysis of traditional chinese medicine syndrome efficacy and significant efficacy rates at Day 7 (per‐protocol set).

	LHQK	Control	Statistic	*p*	Method
N (Nmiss)	64 (0)	64 (0)	NA	0.058	Fisher
Effective, *n* (%)	64 (100.0)	59 (92.2)			
Ineffective, *n* (%)	0 (0.0)	5 (7.8)			
N (Nmiss)	64 (0)	64 (0)	6.000	0.014	Chisq
Significantly effective, *n* (%)	54 (84.4)	42 (65.6)			
Not significantly effective, *n* (%)	10 (15.6)	22 (34.4)			

*Note:* Data are presented as a number (percentage). Efficacy was defined as a ≥ 50% decrease in the total TCM syndrome score. Significant efficacy denotes a more pronounced improvement. Between‐group comparisons were performed using the χ^2^ test or Fisher's exact test, as appropriate.

Abbreviations: Chisq, chi‐square test; LHQK, Lianhua Qingke; NA, not applicable; Nmiss, number of missing values; TCM, traditional Chinese medicine.

### 3.5 Improvement in Laboratory Parameters

There were no significant differences in laboratory parameters, baseline CRP, white blood cell (WBC) counts, or neutrophil percentages (NEUT%) between groups at baseline or Day 7 (*p* > 0.05; Tables 7, 8, 9), highlighting LHQK's symptomatic focus rather than biomarker discovery.

**TABLE 7:** Analysis of C‐reactive protein (CRP) levels (per‐protocol set).

Visit	LHQK	Control	Statistic	*p*	Method
Baseline					
N (Nmiss)	60 (4)	63 (1)	0.397	0.692	*t*‐Test
Mean ± SD	12.3888 ± 11.9927	11.4138 ± 15.0261			
Median	8.6400	6.2700			
95% CI	9.2908, 15.4869	7.6295, 15.1981			
Min–max	0.39–50.6	0.1–68.53			
7‐Day					
N (Nmiss)	57 (7)	59 (5)	−0.150	0.881	*t*‐Test
Mean ± SD	2.5226 ± 2.7138	2.6288 ± 4.6892			
Median	1.1000	0.6800			
95% CI	1.8025, 3.2427	1.4068, 3.8508			
Min–max	0.2–10	0–31.9			
Paired statistic/*p*‐value	−6.214/< 0.001	−3.928/< 0.001			
7‐Day‐baseline					
N (Nmiss)	54 (10)	58 (6)	−1.013	0.313	*t*‐Test
Mean ± SD	−9.4345 ± 11.1564	−7.0376 ± 13.6446			
Median	−6.5150	−3.7150			
95% CI	−12.4796, −6.3893	−10.6253, −3.4499			
Min–max	−48.71–5.56	−58.53–31.26			

*Note:* Data are presented as mean ± standard deviation, median, and range. Between‐group comparisons at each time point and for changes from baseline were performed using the independent *t*‐test or *t*′‐test (for unequal variances), as appropriate. Paired *t*‐tests were used for within‐group comparisons.

Abbreviations: CI, confidence interval; CRP, C‐reactive protein; LHQK, Lianhua Qingke; Nmiss, number of missing values; SD, standard deviation.

**TABLE 8:** Analysis of white blood cell count (per‐protocol set).

Visit	LHQK	Control	Statistic	*p*	Method
Baseline					
N (Nmiss)	62 (2)	63 (1)	0.046	0.964	*t*‐Test
Mean ± SD	8.110 ± 3.415	8.082 ± 3.530			
Median	7.280	7.430			
95% CI	7.243, 8.977	7.193, 8.971			
Min–max	3.52–23.05	3.29–24.7			
7 day					
N (Nmiss)	59 (5)	59 (5)	0.843	0.401	*t*‐Test
Mean ± SD	7.967 ± 2.420	7.567 ± 2.725			
Median	7.760	7.670			
95% CI	7.336, 8.598	6.857, 8.277			
Min–max	4.01–13.9	2.5–15.14			
Paired statistic/*p*‐value	−0.587/0.559	−0.860/0.394			
7 day‐baseline					
N (Nmiss)	57 (7)	58 (6)	0.227	0.821	*t*‐Test
Mean±SD	−0.299 ± 3.844	−0.469 ± 4.151			
Median	−0.050	−0.740			
95% CI	−1.319, 0.721	−1.560, 0.623			
Min–max	−15.68–6.64	−15.29–10.88			

*Note:* Data are presented as mean ± standard deviation, median, and range. Between‐group comparisons at each time point and for changes from baseline were performed using the independent *t*‐test. Paired *t*‐tests were used for within‐group comparisons.

Abbreviations: CI, confidence interval; LHQK, Lianhua Qingke; Nmiss, number of missing values; SD, standard deviation.

**TABLE 9:** Analysis of neutrophil percentage (per‐protocol set).

Visit	LHQK	Control	Statistic	*p*	Method
Baseline					
N (Nmiss)	62 (2)	63 (1)	−0.484	0.629	*t*‐Test
Mean±SD	59.971 ± 11.897	60.983 ± 11.470			
Median	60.500	61.900			
95% CI	56.950, 62.992	58.095, 63.872			
Min–max	28.1–87.7	39.7–91.3			
7‐Day					
N (Nmiss)	59 (5)	59 (5)	0.204	0.838	*t*‐Test
Mean±SD	47.183 ± 9.713	46.792 ± 11.059			
Median	46.700	46.600			
95% CI	44.652, 49.714	43.909, 49.674			
Min–Max	20.6–76.2	24–70.9			
Paired statistic/*p*‐value	−6.621/< 0.001	−7.673/< 0.001			
7‐Day‐baseline					
N (Nmiss)	57 (7)	58 (6)	0.507	0.613	*t*‐Test
Mean ± SD	−13.174 ± 15.021	−14.566 ± 14.457			
Median	−13.900	−14.500			
95% CI	−17.159, −9.188	−18.367, −10.765			
Min–max	−49.2–29.5	−46–16.7			

*Note:* Data are presented as mean ± standard deviation, median, and range. Between‐group comparisons at each time point and for changes from baseline were performed using the independent *t*‐test. Paired *t*‐tests were used for within‐group comparisons.

Abbreviations: CI, confidence interval; LHQK, Lianhua Qingke; Nmiss, number of missing values; SD, standard deviation.

### 3.6 Safety

Adverse events occurred in 2.5% of the LHQK group (two events) versus 8.8% of the placebo group (seven events; *p* > 0.05; Table 10). There were no severe adverse events or progression to critical MPP (Table 11), confirming LHQK's safety. In the LHQK group, the two events were an elevated red blood cell count (*n* = 1) and positive urine leukocytes (*n* = 1). In the placebo group, the seven events included elevated platelet count (*n* = 4), decreased serum creatinine (*n* = 2), elevated alkaline phosphatase (*n* = 1), decreased hemoglobin (*n* = 1), and decreased blood urea (*n* = 1). All adverse events were assessed by the investigators as unrelated to the study medication. No serious adverse events or adverse drug reactions occurred. No patient withdrew due to adverse events. Laboratory safety parameters (complete blood count, liver and kidney function) showed no clinically meaningful differences between groups at baseline or Day 7 (Tables 7, 8, 9), and no patient developed clinically significant laboratory abnormalities requiring intervention. Detailed individual adverse event listings are provided in Table S2.

**TABLE 10:** Adverse events in the safety analysis set (all randomized patients).

	LHQK (*n* = 80)	Control (*n* = 80)	*p*
TEAE *n* (%)	2 (2.5)	7 (8.8)	0.167
ADR *n* (%)	0 (0.0)	0 (0.0)	NA
SAE *n* (%)	0 (0.0)	0 (0.0)	NA
SADR *n* (%)	0 (0.0)	0 (0.0)	NA
Withdrawal	0 (0.0)	0 (0.0)	NA
Death	0 (0.0)	0 (0.0)	NA

*Note:* Data are presented as the number of patients (percentage). TEAE: treatment‐emergent adverse event; ADR: adverse drug reaction; SAE: serious adverse event; SADR: suspected adverse drug reaction. Between‐group comparison of TEAE incidence was performed using the χ^2^ test. All adverse events were assessed as not related to the study medication by the investigators.

Abbreviations: ADR, adverse drug reaction; LHQK, Lianhua Qingke; NA, not applicable; SAE, serious adverse event; SADR, suspected adverse drug reaction; TEAE, treatment‐emergent adverse event.

**TABLE 11:** Analysis of disease progression to severe or critical state (per‐protocol set).

	LHQK	Control	Statistic	*p*	Method
N (Nmiss)	64 (0)	64 (0)	NA	NA	NA
Progression to severe disease, *n* (%)	0 (0.0)	0 (0.0)			
No progression, *n* (%)	64 (100.0)	64 (100.0)			

*Note:* No patients in either group progressed to severe or critical disease during the study period. Statistical comparison was therefore not applicable (NA).

Abbreviations: LHQK, Lianhua Qingke; NA, not applicable; Nmiss, number of missing values.

## 4 Discussion

MP is an atypical pathogen lacking a cell wall, rendering it resistant to cell wall‐targeting antibiotics [8]. Macrolide resistance is prevalent, especially in East Asia, leading to poorer outcomes in children, including prolonged illness and severe complications [3, 9].

In this study, macrolide resistance testing identified the A2063G mutation in 37.6% of tested MP‐positive patients, with 20 cases in the LHQK group and 18 in the control group. While this rate is lower than the 70.0%–90.0% frequently reported in Chinese children, it is consistent with the expected lower resistance prevalence in mild outpatient MPP cases, as resistant strains are known to cause more severe disease and are therefore overrepresented in hospitalized cohorts. Despite this relatively lower resistance rate, adjunctive LHQK therapy significantly improved cough and sputum resolution compared to the placebo. Moreover, given that resistant strains are more likely to cause refractory symptoms, the observed benefit of LHQK in a predominantly susceptible population suggests potential utility even in resistant cases, although this requires direct confirmation in future studies.

MPP presents with fever and cough, often persisting post‐pathogen clearance, increasing risks like plastic bronchitis [10, 11, 12]. Effective management requires symptom relief alongside antimicrobials.

TCM views MPP as “pneumonia with wheezing” due to wind‐heat or phlegm‐heat lung obstruction [13]. LHQK tablets contain 15 herbs, including Ephedra, Gypsum, Mulberry root bark, *Scutellaria baicalensis*, *F. suspensa* , bitter almond, Pinellia, Peucedanum, *Zhejiang fritillaria*, Burdock seed, Rhubarb, Honeysuckle, Platycodonis Radix, tangerine peel, and Glycyrrhiza. Bioactive compounds include kaempferol, quercetin, naringin, luteolin, and β‐sitosterol. Preclinical studies have shown that kaempferol and quercetin modulate cytokine expression (interleukin [IL]‐1β, tumor necrosis factor‐α, IL‐6, IL‐8), thereby alleviating inflammatory responses [14]. Naringin has been demonstrated to suppress NLR family pyrin domain‐containing 3 (NLRP3) inflammasome activation and reduce lung injury in animal models [15]. Luteolin can decrease nuclear factor kappa B (NF‐κB), IL‐17, and IL‐23 levels while increasing peroxisome proliferator‐activated receptor gamma, contributing to immunomodulation [16]. β‐Sitosterol interferes with multiple signaling pathways involved in inflammation, apoptosis, and proliferation without significant toxicity [17].

LHQK alleviates airway injury in viral pneumonia, reduces sputum, promotes ciliary movement, and inhibits NF‐κB/NLRP3 pathways in chronic obstructive pulmonary disease, regulating neutrophil traps and pyroptosis [18, 19, 20, 21].

In this study, the median time to symptom resolution was 4.5 days in the LHQK group versus 5.0 days in the control group, a difference that did not reach statistical significance (*p* = 0.072); however, the proportion of patients achieving symptom resolution by Day 7 was significantly higher in the LHQK group (85.9% vs. 65.6%, *p* < 0.05). Improvements were significant in cough (48.4% vs. 29.7% resolved), sputum (65.6% vs. 40.6%), and throat pain (92.3% vs. 73.3%), but not in fever or headache. Although the 0.5‐day difference in median time to resolution did not reach statistical significance, the absolute difference is modest and should be interpreted in the context of mild, self‐limiting MPP. The more clinically meaningful finding was the significantly higher proportion of patients achieving symptom resolution by Day 7 in the LHQK group (85.9% vs. 65.6%).

Median time to resolution is only one dimension of treatment efficacy. The absolute improvement of 20.3% in the symptom resolution rate at Day 7 (85.9% vs. 65.6%) represents a clinically meaningful benefit. From a clinical and parental perspective, a higher probability of symptom relief by Day 7 may be more important than a modest reduction in median time. Given the excellent safety profile of LHQK (2.5% adverse events, all mild, no serious events, and no drug‐related withdrawals), many parents might accept an additional low‐risk medication if it substantially increases their child's chance of being symptom‐free by the end of the first week. Whether this justifies the addition of another medication is ultimately a shared decision between clinicians and parents, taking into account the child's symptom burden and parental preference. Future pharmacoeconomic studies could further inform this question.

LHQK targets respiratory symptoms via 10 herbs for cough suppression and phlegm resolution (*Peucedanum*, *Platycodon*, *Zhejiang fritillaria*, *Pinellia*, *Ephedra*, bitter almond, mulberry root bark, tangerine peel, burdock seed, and *Glycyrrhiza*), and five herbs for fever reduction (*Scutellaria baicalensis*, Gypsum, Honeysuckle, *Forsythia*, and Rhubarb). Mechanisms include reducing sputum via anti‐inflammatory effects (e.g., *Scutellaria*, Honeysuckle, *Forsythia*) and secretion inhibition (e.g., tangerine peel, *Pinellia*); decreasing viscosity through *Fritillaria*'s glandular stimulation; and promoting expulsion via *Ephedra* (bronchodilation, ciliary enhancement) and *Platycodon* [22, 23, 24]. No differences in CRP, WBC, or NEUT% were noted, but TCM syndrome scores improved significantly in the LHQK group, aligning with TCM's focus on symptoms rather than laboratory test results. The absence of significant changes in CRP, WBC, or NEUT% between groups indicates that LHQK does not primarily act through systemic anti‐inflammatory pathways. Consistent with TCM principles, its value lies in alleviating respiratory symptoms—particularly cough and sputum—rather than directly treating the underlying infection or inflammation. Therefore, LHQK should be viewed as a symptom‐directed adjunctive therapy for mild MPP, complementary to conventional antibiotic treatment, rather than a substitute for anti‐infective therapy.

Acknowledging the self‐limiting nature of mild MPP is important. In our trial, the control group achieved a symptom resolution rate of 65.6% by Day 7, which likely represents the background rate of spontaneous recovery. The additional 20.3% absolute improvement in the LHQK group (85.9% vs. 65.6%) is therefore unlikely to be explained by spontaneous recovery alone. Moreover, the randomized, double‐blind design ensures that any influence of natural disease course was equally distributed between groups. Thus, the net benefit observed can be reasonably attributed to LHQK. Nevertheless, residual confounding by spontaneous recovery cannot be entirely excluded, and a no‐treatment arm in future studies would provide even stronger evidence.

No severe MPP cases occurred, supporting conservative antibiotic use [9, 25]. Several considerations should be noted when extrapolating our findings beyond the study population. First, our trial exclusively enrolled children with mild MPP who were treated in outpatient or inpatient settings. Patients with moderate‐to‐severe MPP—typically requiring hospitalization due to persistent high fever, extensive radiographic involvement, or respiratory distress—were excluded. In such patients, the underlying inflammatory response is substantially more intense, and the clinical course is often complicated by plastic bronchitis, pleural effusion, or extrapulmonary manifestations. Whether LHQK confers similar symptomatic benefits in these more severe cases remains unknown. Second, hospitalized patients differ from outpatients not only in disease severity but also in the intensity of conventional therapy (e.g., intravenous antibiotics, more frequent use of corticosteroids). The adjunctive effects of LHQK may be modified by these concomitant treatments. For instance, in patients receiving systemic corticosteroids, the anti‐inflammatory contribution of LHQK might be less discernible. Third, the pharmacological mechanisms of LHQK—particularly its modulation of the NLRP3 inflammasome and NF‐κB pathways—suggest potential benefit in severe inflammation. However, in the absence of direct evidence, we cannot assume that the magnitude of benefit observed in mild MPP would translate to severe disease. Future trials specifically targeting moderate‐to‐severe MPP, including hospitalized populations, are warranted to address these questions. Safety was favorable, with no differences in liver/kidney function or adverse events between groups (*n* = 80 each). Although the adverse event rate was numerically higher in the placebo group (8.8%) than in the LHQK group (2.5%), the difference was not statistically significant (*p* = 0.167). All events were mild and assessed as unrelated to the study medication. The slightly higher rate in the placebo group is likely attributable to chance variation, given the small number of events and the lack of a dose–response relationship. Moreover, these laboratory abnormalities are commonly observed in pediatric populations as transient fluctuations and were not associated with any clinical symptoms or need for intervention. Importantly, no patient in either group experienced a serious adverse event or required treatment discontinuation. Therefore, the observed imbalance does not raise a safety concern for LHQK.

Symptom‐based endpoints inevitably carry a degree of subjectivity. To minimize this potential bias, we implemented several measures: (i) use of a standardized, protocol‐defined scoring scale; (ii) assessment by trained pediatricians rather than parents; (iii) centralized training and regular monitoring across all 13 centers; and (iv) double‐blinding of both participants and investigators. Nevertheless, some residual variability in symptom interpretation across centers cannot be completely excluded. However, the statistically significant and clinically meaningful differences observed between the LHQK and placebo groups, particularly for cough and sputum, suggest that the treatment effect is robust despite this inherent limitation.

In conclusion, LHQK tablets safely and significantly improve cough and sputum symptoms in children with MPP.

## 5 Limitations

This study has several limitations. Enrollment was restricted to mild MPP cases, limiting generalizability to moderate or severe cases, though it reflects typical “walking pneumonia.” The 7‐day treatment duration prevented assessment of long‐term outcomes like persistent cough or recurrence. Sample size assumptions may have underestimated needs, reducing statistical power for secondary endpoints. Larger, multicenter trials with extended follow‐up are needed to validate LHQK's efficacy across MPP severities. All secondary endpoints were exploratory in nature, and no multiplicity adjustment was applied. Therefore, these findings should be interpreted as hypothesis‐generating and require confirmation in future studies. Although 32 patients (20% of the randomized population) were excluded from the efficacy analysis due to negative central laboratory confirmation, this exclusion was prespecified in the protocol to avoid diagnostic misclassification bias, given the known false‐positive rate of rapid MP tests. The exclusion was balanced between groups (16 in each group), and sensitivity analyses showed no significant baseline differences between included and excluded patients (Table S1), suggesting that selection bias is unlikely. Nevertheless, the exclusion of randomized patients may theoretically affect generalizability, and our findings should be interpreted in the context of this limitation. Finally, the macrolide resistance rate in our mild MPP cohort (37.6%) was lower than the national average (70%–90%), likely because resistant strains often cause more severe disease. This limits generalizability to severe MPP. However, resistant cases were well balanced between groups (20 in LHQK vs. 18 in control), supporting the validity of our primary conclusion for mild MPP. Future studies should assess LHQK's efficacy specifically in resistant populations.

## 6 Conclusion

In this randomized controlled trial conducted in children with mild MPP managed in outpatient and inpatient settings, 7‐day adjunctive LHQK therapy was safe and significantly improved the resolution of cough and sputum in children with mild MPP, leading to a higher symptom resolution rate by Day 7. A trend toward shorter median recovery time was also observed, though it did not reach statistical significance. As an adjunctive therapy, LHQK offers valuable, low‐risk symptomatic relief amid rising macrolide resistance, rather than directly treating the underlying infection. Further research should explore its benefits in severe cases and long‐term scenarios.

**Field‐Specific Terms**
Adverse events: Unfavorable or unintended signs, symptoms, or diseases occurring during a clinical trial, assessed for safety
Baseline characteristics: Initial patient attributes (e.g., age, symptoms) measured at the start of a study to ensure groups are comparable
Bronchodilation: The widening of airways in the lungs, often achieved by medications to improve breathing and reduce cough
Ciliary movement: The coordinated beating of cilia (hair‐like structures) in the respiratory tract, which aids mucus clearance and pathogen removal
Community‐acquired pneumonia: Pneumonia contracted outside of a hospital setting, often caused by pathogens like *Mycoplasma pneumoniae*
Compliance: Adherence to the study protocol, such as taking medications as prescribed, is measured to ensure treatment fidelity
Cytokines: Signaling proteins (e.g., IL‐1β, TNF‐α) released by immune cells that regulate inflammation and immune responses
Double‐blind: A study design where neither participants nor researchers know the treatment assignments, reducing bias
Efficacy: The ability of an intervention (e.g., LHQK) to produce a desired therapeutic effect, measured by endpoints like symptom resolution
Fisher's exact test: A statistical test for analyzing contingency tables, especially with small sample sizes, to assess associations
Kaplan–Meier analysis: A statistical method for estimating survival or event‐free probabilities over time; used for symptom resolution rates
Log‐rank test: A statistical test comparing survival curves from Kaplan–Meier analysis to detect differences between groups
Macrolide resistance: Reduced effectiveness of macrolide antibiotics (e.g., azithromycin) against bacteria like *Mycoplasma pneumoniae* due to genetic changes
NF‐κB: nuclear factor kappa B – A protein complex that regulates genes involved in inflammation and immune responses
NLRP3 inflammasome: A multiprotein complex that activates inflammatory pathways, contributing to conditions like lung injury
*p*‐value: The probability of observing results as extreme as those in the data, assuming no true difference; *P* < 0.05 indicates statistical significance
Phlegm‐heat obstructing the lung: A Traditional Chinese Medicine pattern describing lung congestion with heat and mucus, leading to cough and sputum
Placebo‐controlled trial: A study comparing an active treatment to an inactive placebo to isolate the treatment's effects
Plastic bronchitis: A rare complication of respiratory infections where mucus forms casts in the airways, potentially causing breathing difficulties
Primary endpoint: The main outcome measure in a study (e.g., symptom resolution rate) used to assess efficacy
Pyroptosis: A form of programmed cell death triggered by inflammation, often involving immune cells like neutrophils
Randomized Controlled Trial: A study design where participants are randomly assigned to treatment groups to minimize bias
Secondary endpoints: Additional outcome measures (e.g., individual symptom relief) supporting the primary endpoint
Symptom resolution rate: The proportion of participants achieving a predefined improvement in symptoms (e.g., ≥ 1‐level reduction) within a timeframe
TCM syndrome score: A scoring system in Traditional Chinese Medicine quantifying symptom patterns (e.g., phlegm‐heat) for efficacy assessment
Wilcoxon rank‐sum test: A non‐parametric statistical test comparing two independent groups for differences in medians or distributions
Wind‐heat invading the lungs: A Traditional Chinese Medicine pattern of acute respiratory infection with fever and cough due to external heat invasion
Withdrawal criteria: Conditions under which participants are removed from a study (e.g., adverse events or non‐compliance) to protect safety

## Author Contributions

**He Wang:** Conceptualization, Methodology, Formal Analysis, Writing – Original Draft, Project Administration, Data Curation, Validation, Visualization. **Haiwei Dou:** Investigation, Data Curation, Validation. **Guangying Chen**, **Yanmin Wei**, **Hua Li**, **Shuqiong Xu**, **Rongzhen Kang**, **Shanhong Liu**, **Long Zhang**, **Nan Li**, **Hongli Sun**, **Chi Wang**, **Jie Zhao**, **Jirong Yi**, **Wentao Song,** and **Hongrong Li:** Investigation, Resources. **Rong Ma** and **Kunling Shen:** Conceptualization, Methodology, Supervision, Writing – Review and Editing. **Deli Xin** and **Wenjie Qi:** Validation, Formal Analysis, Conceptualization, Methodology, Supervision, Funding Acquisition, Writing – Review and Editing.

## Funding

The study was funded by the National Key Clinical Specialty Discipline Construction Program of China (Document No. [2022] 468 issued by the Medical Administration Bureau of National Health Commission & Document No. [2023] 2 issued by the Medical Quality Office of National Health Commission), and the Program for Cultivating High‐Level Public Health Technical Talents (Academic Discipline Leader Category, Grant No. 02‐29).

## Ethics Statement

This study was conducted in accordance with the Declaration of Helsinki. The protocol was approved by the ethics committees of all participating centers. Written informed consent was obtained from the guardians of all participants. The trial was registered in the Chinese Clinical Trial Registry (ChiCTR2300078209) on November 30, 2023, prior to the enrollment of the first patient.

## Conflicts of Interest

The authors declare no conflicts of interest.

## Supporting information


**Data S1:** Supporting Information.

**Data S2:** Supporting Information.

**Data S3:** Supporting Information.

**Table S1:** Baseline characteristics of included (MPP, *n* = 128) vs. excluded (non‐MPP, *n* = 32) patients.

**Table S2:** Detailed listing of treatment‐emergent adverse events (safety set).

## Data Availability

The datasets used and/or analyzed in this study are available from the corresponding author upon reasonable request.
