# Supplementary material for: Efficacy and Safety of Lianhua Qingke Tablets in Children With Mycoplasma pneumoniae Pneumonia: A Randomized, Double‐Blind, Multicenter, Placebo‐Controlled Clinical Trial
Source: Clin Respir J. 2026 Jun 25;20(7):e70204. doi: 10.1111/crj.70204 (PMC13304230; doi:10.1111/crj.70204)
Supplement: Supplementary file 1 — Data S1: Supporting Information. [file CRJ-20-e70204-s005.docx]

列表1-1 试验完成情况列表(随机入组受试者)

| 组别 | 中心 | 随机号 | 完成试验 | 退出研究日期 | 退出原因 | 是否破盲 |
| --- | --- | --- | --- | --- | --- | --- |
| 试验组 | K01 | 002 | 是 |  |  |  |
| 试验组 | K01 | 003 | 是 |  |  |  |
| 试验组 | K01 | 005 | 是 |  |  |  |
| 试验组 | K01 | 007 | 是 |  |  |  |
| 试验组 | K01 | 057 | 是 |  |  |  |
| 试验组 | K01 | 060 | 否 | 2024-01-11 | 依从性差: 拒绝出组检查，未查 | 否 |
| 试验组 | K01 | 111 | 是 |  |  |  |
| 试验组 | K01 | 113 | 是 |  |  |  |
| 试验组 | K01 | 137 | 是 |  |  |  |
| 试验组 | K01 | 138 | 是 |  |  |  |
| 试验组 | K01 | 178 | 是 |  |  |  |
| 试验组 | K01 | 179 | 是 |  |  |  |
| 试验组 | K02 | 018 | 是 |  |  |  |
| 试验组 | K02 | 020 | 是 |  |  |  |
| 试验组 | K02 | 021 | 是 |  |  |  |
| 试验组 | K02 | 022 | 是 |  |  |  |
| 试验组 | K02 | 025 | 是 |  |  |  |
| 试验组 | K02 | 027 | 是 |  |  |  |
| 试验组 | K02 | 029 | 是 |  |  |  |
| 试验组 | K02 | 030 | 是 |  |  |  |
| 试验组 | K05 | 048 | 是 |  |  |  |
| 试验组 | K05 | 050 | 是 |  |  |  |
| 试验组 | K05 | 056 | 是 |  |  |  |
| 试验组 | K05 | 095 | 是 |  |  |  |
| 试验组 | K06 | 081 | 是 |  |  |  |
| 试验组 | K06 | 082 | 是 |  |  |  |
| 试验组 | K06 | 087 | 是 |  |  |  |
| 试验组 | K06 | 088 | 是 |  |  |  |
| 试验组 | K06 | 090 | 是 |  |  |  |
| 试验组 | K06 | 092 | 是 |  |  |  |
| 试验组 | K06 | 150 | 是 |  |  |  |
| 试验组 | K06 | 152 | 是 |  |  |  |
| 试验组 | K06 | 154 | 是 |  |  |  |
| 试验组 | K06 | 155 | 是 |  |  |  |
| 试验组 | K06 | 203 | 是 |  |  |  |
| 试验组 | K06 | 205 | 是 |  |  |  |
| 试验组 | K06 | 206 | 是 |  |  |  |
| 试验组 | K06 | 209 | 是 |  |  |  |
| 试验组 | K06 | 210 | 是 |  |  |  |
| 试验组 | K06 | 229 | 是 |  |  |  |
| 试验组 | K07 | 061 | 是 |  |  |  |
| 试验组 | K07 | 062 | 是 |  |  |  |
| 试验组 | K07 | 066 | 是 |  |  |  |
| 试验组 | K07 | 067 | 是 |  |  |  |
| 试验组 | K09 | 122 | 是 |  |  |  |
| 试验组 | K09 | 123 | 是 |  |  |  |
| 试验组 | K09 | 126 | 是 |  |  |  |
| 试验组 | K09 | 127 | 是 |  |  |  |
| 试验组 | K09 | 157 | 是 |  |  |  |
| 试验组 | K09 | 159 | 是 |  |  |  |
| 试验组 | K10 | 103 | 是 |  |  |  |
| 试验组 | K10 | 116 | 是 |  |  |  |
| 试验组 | K10 | 117 | 是 |  |  |  |
| 试验组 | K10 | 119 | 是 |  |  |  |
| 试验组 | K10 | 189 | 是 |  |  |  |
| 试验组 | K10 | 191 | 是 |  |  |  |
| 试验组 | K10 | 195 | 是 |  |  |  |
| 试验组 | K10 | 196 | 是 |  |  |  |
| 试验组 | K10 | 197 | 是 |  |  |  |
| 试验组 | K10 | 199 | 是 |  |  |  |
| 试验组 | K10 | 202 | 是 |  |  |  |
| 试验组 | K12 | 169 | 是 |  |  |  |
| 试验组 | K13 | 141 | 是 |  |  |  |
| 试验组 | K13 | 143 | 是 |  |  |  |
| 试验组 | K13 | 146 | 是 |  |  |  |
| 试验组 | K14 | 185 | 是 |  |  |  |
| 试验组 | K14 | 187 | 是 |  |  |  |
| 试验组 | K14 | 235 | 是 |  |  |  |
| 试验组 | K14 | 238 | 是 |  |  |  |
| 试验组 | K14 | 240 | 是 |  |  |  |
| 试验组 | K15 | 010 | 是 |  |  |  |
| 试验组 | K15 | 011 | 是 |  |  |  |
| 试验组 | K15 | 013 | 是 |  |  |  |
| 试验组 | K15 | 015 | 是 |  |  |  |
| 试验组 | K15 | 219 | 是 |  |  |  |
| 试验组 | K15 | 221 | 是 |  |  |  |
| 试验组 | K15 | 223 | 是 |  |  |  |
| 试验组 | K15 | 225 | 是 |  |  |  |
| 试验组 | K15 | 226 | 是 |  |  |  |
| 试验组 | K16 | 213 | 是 |  |  |  |
| 对照组 | K01 | 001 | 是 |  |  |  |
| 对照组 | K01 | 004 | 是 |  |  |  |
| 对照组 | K01 | 006 | 是 |  |  |  |
| 对照组 | K01 | 008 | 是 |  |  |  |
| 对照组 | K01 | 058 | 是 |  |  |  |
| 对照组 | K01 | 059 | 是 |  |  |  |
| 对照组 | K01 | 112 | 是 |  |  |  |
| 对照组 | K01 | 114 | 是 |  |  |  |
| 对照组 | K01 | 139 | 是 |  |  |  |
| 对照组 | K01 | 140 | 是 |  |  |  |
| 对照组 | K01 | 177 | 是 |  |  |  |
| 对照组 | K01 | 180 | 是 |  |  |  |
| 对照组 | K02 | 017 | 是 |  |  |  |
| 对照组 | K02 | 019 | 是 |  |  |  |
| 对照组 | K02 | 023 | 是 |  |  |  |
| 对照组 | K02 | 024 | 是 |  |  |  |
| 对照组 | K02 | 026 | 是 |  |  |  |
| 对照组 | K02 | 028 | 是 |  |  |  |
| 对照组 | K03 | 037 | 是 |  |  |  |
| 对照组 | K05 | 047 | 是 |  |  |  |
| 对照组 | K05 | 049 | 是 |  |  |  |
| 对照组 | K05 | 051 | 是 |  |  |  |
| 对照组 | K05 | 053 | 是 |  |  |  |
| 对照组 | K05 | 055 | 是 |  |  |  |
| 对照组 | K05 | 093 | 是 |  |  |  |
| 对照组 | K05 | 094 | 是 |  |  |  |
| 对照组 | K06 | 083 | 是 |  |  |  |
| 对照组 | K06 | 084 | 是 |  |  |  |
| 对照组 | K06 | 085 | 是 |  |  |  |
| 对照组 | K06 | 086 | 是 |  |  |  |
| 对照组 | K06 | 089 | 是 |  |  |  |
| 对照组 | K06 | 091 | 是 |  |  |  |
| 对照组 | K06 | 149 | 是 |  |  |  |
| 对照组 | K06 | 151 | 是 |  |  |  |
| 对照组 | K06 | 153 | 是 |  |  |  |
| 对照组 | K06 | 156 | 是 |  |  |  |
| 对照组 | K06 | 204 | 是 |  |  |  |
| 对照组 | K06 | 207 | 是 |  |  |  |
| 对照组 | K06 | 208 | 是 |  |  |  |
| 对照组 | K06 | 227 | 是 |  |  |  |
| 对照组 | K06 | 228 | 是 |  |  |  |
| 对照组 | K07 | 063 | 是 |  |  |  |
| 对照组 | K07 | 064 | 是 |  |  |  |
| 对照组 | K07 | 065 | 是 |  |  |  |
| 对照组 | K07 | 068 | 是 |  |  |  |
| 对照组 | K07 | 069 | 是 |  |  |  |
| 对照组 | K07 | 070 | 是 |  |  |  |
| 对照组 | K09 | 121 | 是 |  |  |  |
| 对照组 | K09 | 124 | 是 |  |  |  |
| 对照组 | K09 | 125 | 是 |  |  |  |
| 对照组 | K09 | 128 | 是 |  |  |  |
| 对照组 | K09 | 158 | 是 |  |  |  |
| 对照组 | K09 | 160 | 是 |  |  |  |
| 对照组 | K09 | 161 | 是 |  |  |  |
| 对照组 | K10 | 115 | 是 |  |  |  |
| 对照组 | K10 | 118 | 是 |  |  |  |
| 对照组 | K10 | 120 | 是 |  |  |  |
| 对照组 | K10 | 190 | 是 |  |  |  |
| 对照组 | K10 | 192 | 是 |  |  |  |
| 对照组 | K10 | 193 | 是 |  |  |  |
| 对照组 | K10 | 194 | 是 |  |  |  |
| 对照组 | K10 | 198 | 是 |  |  |  |
| 对照组 | K10 | 200 | 是 |  |  |  |
| 对照组 | K10 | 201 | 是 |  |  |  |
| 对照组 | K13 | 142 | 是 |  |  |  |
| 对照组 | K13 | 148 | 是 |  |  |  |
| 对照组 | K14 | 183 | 是 |  |  |  |
| 对照组 | K14 | 184 | 是 |  |  |  |
| 对照组 | K14 | 186 | 是 |  |  |  |
| 对照组 | K14 | 236 | 是 |  |  |  |
| 对照组 | K14 | 237 | 是 |  |  |  |
| 对照组 | K14 | 239 | 是 |  |  |  |
| 对照组 | K15 | 009 | 是 |  |  |  |
| 对照组 | K15 | 012 | 是 |  |  |  |
| 对照组 | K15 | 014 | 是 |  |  |  |
| 对照组 | K15 | 220 | 是 |  |  |  |
| 对照组 | K15 | 222 | 是 |  |  |  |
| 对照组 | K15 | 224 | 是 |  |  |  |
| 对照组 | K16 | 211 | 是 |  |  |  |
| 对照组 | K16 | 212 | 是 |  |  |  |

列表1-2 受试者人群划分详细列表(随机入组受试者)

| 组别 | 中心 | 随机号 | FAS | MPP | PPS | SS | 未入PPS原因 |
| --- | --- | --- | --- | --- | --- | --- | --- |
| 试验组 | K01 | 002 | Y | Y | Y | Y |  |
| 试验组 | K01 | 003 | Y | Y | Y | Y |  |
| 试验组 | K01 | 005 | Y | Y | Y | Y |  |
| 试验组 | K01 | 007 | Y | Y | Y | Y |  |
| 试验组 | K01 | 057 | Y | Y | Y | Y |  |
| 试验组 | K01 | 060 | Y | N | N | Y | 受试者拒绝出组检查 |
| 试验组 | K01 | 111 | Y | Y | Y | Y |  |
| 试验组 | K01 | 113 | Y | Y | Y | Y |  |
| 试验组 | K01 | 137 | Y | Y | Y | Y |  |
| 试验组 | K01 | 138 | Y | Y | Y | Y |  |
| 试验组 | K01 | 178 | Y | N | Y | Y |  |
| 试验组 | K01 | 179 | Y | Y | Y | Y |  |
| 试验组 | K02 | 018 | Y | Y | Y | Y |  |
| 试验组 | K02 | 020 | Y | N | Y | Y |  |
| 试验组 | K02 | 021 | Y | N | Y | Y |  |
| 试验组 | K02 | 022 | Y | Y | Y | Y |  |
| 试验组 | K02 | 025 | Y | Y | Y | Y |  |
| 试验组 | K02 | 027 | Y | Y | Y | Y |  |
| 试验组 | K02 | 029 | Y | Y | Y | Y |  |
| 试验组 | K02 | 030 | Y | Y | Y | Y |  |
| 试验组 | K05 | 048 | Y | Y | Y | Y |  |
| 试验组 | K05 | 050 | Y | Y | Y | Y |  |
| 试验组 | K05 | 056 | Y | N | Y | Y |  |
| 试验组 | K05 | 095 | Y | Y | Y | Y |  |
| 试验组 | K06 | 081 | Y |  | Y | Y |  |
| 试验组 | K06 | 082 | Y | N | Y | Y |  |
| 试验组 | K06 | 087 | Y | Y | Y | Y |  |
| 试验组 | K06 | 088 | Y | Y | Y | Y |  |
| 试验组 | K06 | 090 | Y | Y | Y | Y |  |
| 试验组 | K06 | 092 | Y | Y | Y | Y |  |
| 试验组 | K06 | 150 | Y | Y | Y | Y |  |
| 试验组 | K06 | 152 | Y | Y | Y | Y |  |
| 试验组 | K06 | 154 | Y | Y | Y | Y |  |
| 试验组 | K06 | 155 | Y | N | Y | Y |  |
| 试验组 | K06 | 203 | Y | Y | Y | Y |  |
| 试验组 | K06 | 205 | Y | Y | Y | Y |  |
| 试验组 | K06 | 206 | Y | Y | Y | Y |  |
| 试验组 | K06 | 209 | Y | Y | Y | Y |  |
| 试验组 | K06 | 210 | Y | Y | Y | Y |  |
| 试验组 | K06 | 229 | Y | Y | Y | Y |  |
| 试验组 | K07 | 061 | Y | Y | Y | Y |  |
| 试验组 | K07 | 062 | Y | Y | Y | Y |  |
| 试验组 | K07 | 066 | Y | Y | Y | Y |  |
| 试验组 | K07 | 067 | Y | Y | Y | Y |  |
| 试验组 | K09 | 122 | Y | Y | Y | Y |  |
| 试验组 | K09 | 123 | Y | Y | Y | Y |  |
| 试验组 | K09 | 126 | Y | Y | Y | Y |  |
| 试验组 | K09 | 127 | Y | N | Y | Y |  |
| 试验组 | K09 | 157 | Y | Y | Y | Y |  |
| 试验组 | K09 | 159 | Y | Y | Y | Y |  |
| 试验组 | K10 | 103 | Y | Y | Y | Y |  |
| 试验组 | K10 | 116 | Y | Y | Y | Y |  |
| 试验组 | K10 | 117 | Y | Y | Y | Y |  |
| 试验组 | K10 | 119 | Y | Y | Y | Y |  |
| 试验组 | K10 | 189 | Y | Y | Y | Y |  |
| 试验组 | K10 | 191 | Y | Y | Y | Y |  |
| 试验组 | K10 | 195 | Y | Y | Y | Y |  |
| 试验组 | K10 | 196 | Y | Y | Y | Y |  |
| 试验组 | K10 | 197 | Y | Y | Y | Y |  |
| 试验组 | K10 | 199 | Y | Y | Y | Y |  |
| 试验组 | K10 | 202 | Y | Y | Y | Y |  |
| 试验组 | K12 | 169 | Y | Y | Y | Y |  |
| 试验组 | K13 | 141 | Y | Y | Y | Y |  |
| 试验组 | K13 | 143 | Y | N | N | Y | 用药依从性为57.14% |
| 试验组 | K13 | 146 | Y | Y | Y | Y |  |
| 试验组 | K14 | 185 | Y | Y | Y | Y |  |
| 试验组 | K14 | 187 | Y | N | Y | Y |  |
| 试验组 | K14 | 235 | Y | N | Y | Y |  |
| 试验组 | K14 | 238 | Y | Y | Y | Y |  |
| 试验组 | K14 | 240 | Y | N | Y | Y |  |
| 试验组 | K15 | 010 | Y | Y | Y | Y |  |
| 试验组 | K15 | 011 | Y | Y | Y | Y |  |
| 试验组 | K15 | 013 | Y | Y | Y | Y |  |
| 试验组 | K15 | 015 | Y | Y | Y | Y |  |
| 试验组 | K15 | 219 | Y | N | Y | Y |  |
| 试验组 | K15 | 221 | Y | Y | Y | Y |  |
| 试验组 | K15 | 223 | Y | N | Y | Y |  |
| 试验组 | K15 | 225 | Y | N | Y | Y |  |
| 试验组 | K15 | 226 | Y | Y | Y | Y |  |
| 试验组 | K16 | 213 | Y | Y | Y | Y |  |
| 对照组 | K01 | 001 | Y | Y | Y | Y |  |
| 对照组 | K01 | 004 | Y | N | Y | Y |  |
| 对照组 | K01 | 006 | Y | Y | Y | Y |  |
| 对照组 | K01 | 008 | Y | Y | Y | Y |  |
| 对照组 | K01 | 058 | Y | Y | Y | Y |  |
| 对照组 | K01 | 059 | Y | Y | Y | Y |  |
| 对照组 | K01 | 112 | Y | Y | Y | Y |  |
| 对照组 | K01 | 114 | Y | Y | Y | Y |  |
| 对照组 | K01 | 139 | Y | N | Y | Y |  |
| 对照组 | K01 | 140 | Y | N | Y | Y |  |
| 对照组 | K01 | 177 | Y | N | Y | Y |  |
| 对照组 | K01 | 180 | Y | N | Y | Y |  |
| 对照组 | K02 | 017 | Y | Y | Y | Y |  |
| 对照组 | K02 | 019 | Y | Y | Y | Y |  |
| 对照组 | K02 | 023 | Y | Y | Y | Y |  |
| 对照组 | K02 | 024 | Y | N | Y | Y |  |
| 对照组 | K02 | 026 | Y | N | Y | Y |  |
| 对照组 | K02 | 028 | Y | N | Y | Y |  |
| 对照组 | K03 | 037 | Y | Y | Y | Y |  |
| 对照组 | K05 | 047 | Y | Y | Y | Y |  |
| 对照组 | K05 | 049 | Y | Y | Y | Y |  |
| 对照组 | K05 | 051 | Y | Y | Y | Y |  |
| 对照组 | K05 | 053 | Y | Y | Y | Y |  |
| 对照组 | K05 | 055 | Y | N | Y | Y |  |
| 对照组 | K05 | 093 | Y | Y | Y | Y |  |
| 对照组 | K05 | 094 | Y | N | Y | Y |  |
| 对照组 | K06 | 083 | Y | N | Y | Y |  |
| 对照组 | K06 | 084 | Y | Y | Y | Y |  |
| 对照组 | K06 | 085 | Y | Y | Y | Y |  |
| 对照组 | K06 | 086 | Y | Y | Y | Y |  |
| 对照组 | K06 | 089 | Y | Y | Y | Y |  |
| 对照组 | K06 | 091 | Y | Y | Y | Y |  |
| 对照组 | K06 | 149 | Y | Y | Y | Y |  |
| 对照组 | K06 | 151 | Y | Y | Y | Y |  |
| 对照组 | K06 | 153 | Y | Y | Y | Y |  |
| 对照组 | K06 | 156 | Y | N | Y | Y |  |
| 对照组 | K06 | 204 | Y | Y | Y | Y |  |
| 对照组 | K06 | 207 | Y | Y | Y | Y |  |
| 对照组 | K06 | 208 | Y | Y | Y | Y |  |
| 对照组 | K06 | 227 | Y | Y | Y | Y |  |
| 对照组 | K06 | 228 | Y | Y | Y | Y |  |
| 对照组 | K07 | 063 | Y | Y | Y | Y |  |
| 对照组 | K07 | 064 | Y | Y | Y | Y |  |
| 对照组 | K07 | 065 | Y | Y | Y | Y |  |
| 对照组 | K07 | 068 | Y | Y | Y | Y |  |
| 对照组 | K07 | 069 | Y | Y | Y | Y |  |
| 对照组 | K07 | 070 | Y | Y | Y | Y |  |
| 对照组 | K09 | 121 | Y | Y | Y | Y |  |
| 对照组 | K09 | 124 | Y | Y | Y | Y |  |
| 对照组 | K09 | 125 | Y | Y | Y | Y |  |
| 对照组 | K09 | 128 | Y | N | Y | Y |  |
| 对照组 | K09 | 158 | Y | Y | Y | Y |  |
| 对照组 | K09 | 160 | Y | Y | Y | Y |  |
| 对照组 | K09 | 161 | Y |  | Y | Y |  |
| 对照组 | K10 | 115 | Y | Y | Y | Y |  |
| 对照组 | K10 | 118 | Y | Y | Y | Y |  |
| 对照组 | K10 | 120 | Y | Y | Y | Y |  |
| 对照组 | K10 | 190 | Y | Y | Y | Y |  |
| 对照组 | K10 | 192 | Y | Y | Y | Y |  |
| 对照组 | K10 | 193 | Y | Y | Y | Y |  |
| 对照组 | K10 | 194 | Y | Y | Y | Y |  |
| 对照组 | K10 | 198 | Y | Y | Y | Y |  |
| 对照组 | K10 | 200 | Y | Y | Y | Y |  |
| 对照组 | K10 | 201 | Y | Y | Y | Y |  |
| 对照组 | K13 | 142 | Y | Y | Y | Y |  |
| 对照组 | K13 | 148 | Y | Y | Y | Y |  |
| 对照组 | K14 | 183 | Y | Y | Y | Y |  |
| 对照组 | K14 | 184 | Y | Y | Y | Y |  |
| 对照组 | K14 | 186 | Y | Y | Y | Y |  |
| 对照组 | K14 | 236 | Y | Y | Y | Y |  |
| 对照组 | K14 | 237 | Y | Y | Y | Y |  |
| 对照组 | K14 | 239 | Y | N | Y | Y |  |
| 对照组 | K15 | 009 | Y | Y | Y | Y |  |
| 对照组 | K15 | 012 | Y | Y | Y | Y |  |
| 对照组 | K15 | 014 | Y | Y | Y | Y |  |
| 对照组 | K15 | 220 | Y | Y | Y | Y |  |
| 对照组 | K15 | 222 | Y | N | Y | Y |  |
| 对照组 | K15 | 224 | Y | Y | Y | Y |  |
| 对照组 | K16 | 211 | Y | Y | Y | Y |  |
| 对照组 | K16 | 212 | Y | Y | Y | Y |  |

列表1-3 终止研究的受试者列表(随机入组受试者)

| 组别 | 中心 | 随机号 | 随机时间 | 退出研究日期 | 退出原因 | 是否服药 | FAS | SS | PPS |
| --- | --- | --- | --- | --- | --- | --- | --- | --- | --- |
| 试验组 | K01 | 002 | 2023-12-09 |  |  | 是 | Y | Y | Y |
| 试验组 | K01 | 003 | 2023-12-11 |  |  | 是 | Y | Y | Y |
| 试验组 | K01 | 005 | 2023-12-16 |  |  | 是 | Y | Y | Y |
| 试验组 | K01 | 007 | 2023-12-26 |  |  | 是 | Y | Y | Y |
| 试验组 | K01 | 057 | 2023-12-28 |  |  | 是 | Y | Y | Y |
| 试验组 | K01 | 060 | 2024-01-05 | 2024-01-11 | 依从性差: 拒绝出组检查，未查 | 是 | Y | Y | N |
| 试验组 | K01 | 111 | 2024-01-06 |  |  | 是 | Y | Y | Y |
| 试验组 | K01 | 113 | 2024-01-20 |  |  | 是 | Y | Y | Y |
| 试验组 | K01 | 137 | 2024-01-20 |  |  | 是 | Y | Y | Y |
| 试验组 | K01 | 138 | 2024-01-24 |  |  | 是 | Y | Y | Y |
| 试验组 | K01 | 178 | 2024-03-11 |  |  | 是 | Y | Y | Y |
| 试验组 | K01 | 179 | 2024-03-12 |  |  | 是 | Y | Y | Y |
| 试验组 | K02 | 018 | 2024-01-10 |  |  | 是 | Y | Y | Y |
| 试验组 | K02 | 020 | 2024-01-19 |  |  | 是 | Y | Y | Y |
| 试验组 | K02 | 021 | 2024-01-23 |  |  | 是 | Y | Y | Y |
| 试验组 | K02 | 022 | 2024-01-24 |  |  | 是 | Y | Y | Y |
| 试验组 | K02 | 025 | 2024-01-25 |  |  | 是 | Y | Y | Y |
| 试验组 | K02 | 027 | 2024-01-30 |  |  | 是 | Y | Y | Y |
| 试验组 | K02 | 029 | 2024-02-05 |  |  | 是 | Y | Y | Y |
| 试验组 | K02 | 030 | 2024-02-24 |  |  | 是 | Y | Y | Y |
| 试验组 | K05 | 048 | 2023-12-20 |  |  | 是 | Y | Y | Y |
| 试验组 | K05 | 050 | 2023-12-28 |  |  | 是 | Y | Y | Y |
| 试验组 | K05 | 056 | 2024-01-22 |  |  | 是 | Y | Y | Y |
| 试验组 | K05 | 095 | 2024-02-29 |  |  | 是 | Y | Y | Y |
| 试验组 | K06 | 081 | 2023-12-22 |  |  | 是 | Y | Y | Y |
| 试验组 | K06 | 082 | 2023-12-27 |  |  | 是 | Y | Y | Y |
| 试验组 | K06 | 087 | 2024-01-07 |  |  | 是 | Y | Y | Y |
| 试验组 | K06 | 088 | 2024-01-09 |  |  | 是 | Y | Y | Y |
| 试验组 | K06 | 090 | 2024-01-13 |  |  | 是 | Y | Y | Y |
| 试验组 | K06 | 092 | 2024-01-14 |  |  | 是 | Y | Y | Y |
| 试验组 | K06 | 150 | 2024-01-20 |  |  | 是 | Y | Y | Y |
| 试验组 | K06 | 152 | 2024-01-21 |  |  | 是 | Y | Y | Y |
| 试验组 | K06 | 154 | 2024-01-27 |  |  | 是 | Y | Y | Y |
| 试验组 | K06 | 155 | 2024-01-27 |  |  | 是 | Y | Y | Y |
| 试验组 | K06 | 203 | 2024-02-03 |  |  | 是 | Y | Y | Y |
| 试验组 | K06 | 205 | 2024-02-19 |  |  | 是 | Y | Y | Y |
| 试验组 | K06 | 206 | 2024-02-20 |  |  | 是 | Y | Y | Y |
| 试验组 | K06 | 209 | 2024-02-23 |  |  | 是 | Y | Y | Y |
| 试验组 | K06 | 210 | 2024-02-23 |  |  | 是 | Y | Y | Y |
| 试验组 | K06 | 229 | 2024-03-18 |  |  | 是 | Y | Y | Y |
| 试验组 | K07 | 061 | 2023-12-23 |  |  | 是 | Y | Y | Y |
| 试验组 | K07 | 062 | 2023-12-26 |  |  | 是 | Y | Y | Y |
| 试验组 | K07 | 066 | 2023-12-28 |  |  | 是 | Y | Y | Y |
| 试验组 | K07 | 067 | 2024-01-04 |  |  | 是 | Y | Y | Y |
| 试验组 | K09 | 122 | 2024-01-11 |  |  | 是 | Y | Y | Y |
| 试验组 | K09 | 123 | 2024-01-11 |  |  | 是 | Y | Y | Y |
| 试验组 | K09 | 126 | 2024-01-16 |  |  | 是 | Y | Y | Y |
| 试验组 | K09 | 127 | 2024-01-16 |  |  | 是 | Y | Y | Y |
| 试验组 | K09 | 157 | 2024-01-18 |  |  | 是 | Y | Y | Y |
| 试验组 | K09 | 159 | 2024-01-18 |  |  | 是 | Y | Y | Y |
| 试验组 | K10 | 103 | 2024-03-10 |  |  | 是 | Y | Y | Y |
| 试验组 | K10 | 116 | 2024-01-16 |  |  | 是 | Y | Y | Y |
| 试验组 | K10 | 117 | 2024-01-24 |  |  | 是 | Y | Y | Y |
| 试验组 | K10 | 119 | 2024-01-27 |  |  | 是 | Y | Y | Y |
| 试验组 | K10 | 189 | 2024-01-29 |  |  | 是 | Y | Y | Y |
| 试验组 | K10 | 191 | 2024-02-06 |  |  | 是 | Y | Y | Y |
| 试验组 | K10 | 195 | 2024-02-20 |  |  | 是 | Y | Y | Y |
| 试验组 | K10 | 196 | 2024-02-21 |  |  | 是 | Y | Y | Y |
| 试验组 | K10 | 197 | 2024-02-27 |  |  | 是 | Y | Y | Y |
| 试验组 | K10 | 199 | 2024-03-03 |  |  | 是 | Y | Y | Y |
| 试验组 | K10 | 202 | 2024-03-09 |  |  | 是 | Y | Y | Y |
| 试验组 | K12 | 169 | 2024-02-26 |  |  | 是 | Y | Y | Y |
| 试验组 | K13 | 141 | 2024-03-19 |  |  | 是 | Y | Y | Y |
| 试验组 | K13 | 143 | 2024-03-19 |  |  | 是 | Y | Y | N |
| 试验组 | K13 | 146 | 2024-03-11 |  |  | 是 | Y | Y | Y |
| 试验组 | K14 | 185 | 2024-03-19 |  |  | 是 | Y | Y | Y |
| 试验组 | K14 | 187 | 2024-03-21 |  |  | 是 | Y | Y | Y |
| 试验组 | K14 | 235 | 2024-03-13 |  |  | 是 | Y | Y | Y |
| 试验组 | K14 | 238 | 2024-03-15 |  |  | 是 | Y | Y | Y |
| 试验组 | K14 | 240 | 2024-03-16 |  |  | 是 | Y | Y | Y |
| 试验组 | K15 | 010 | 2024-03-20 |  |  | 是 | Y | Y | Y |
| 试验组 | K15 | 011 | 2024-03-13 |  |  | 是 | Y | Y | Y |
| 试验组 | K15 | 013 | 2024-03-18 |  |  | 是 | Y | Y | Y |
| 试验组 | K15 | 015 | 2024-03-22 |  |  | 是 | Y | Y | Y |
| 试验组 | K15 | 219 | 2024-02-28 |  |  | 是 | Y | Y | Y |
| 试验组 | K15 | 221 | 2024-03-03 |  |  | 是 | Y | Y | Y |
| 试验组 | K15 | 223 | 2024-03-06 |  |  | 是 | Y | Y | Y |
| 试验组 | K15 | 225 | 2024-03-13 |  |  | 是 | Y | Y | Y |
| 试验组 | K15 | 226 | 2024-03-14 |  |  | 是 | Y | Y | Y |
| 试验组 | K16 | 213 | 2024-03-18 |  |  | 是 | Y | Y | Y |
| 对照组 | K01 | 001 | 2023-12-09 |  |  | 是 | Y | Y | Y |
| 对照组 | K01 | 004 | 2023-12-14 |  |  | 是 | Y | Y | Y |
| 对照组 | K01 | 006 | 2023-12-22 |  |  | 是 | Y | Y | Y |
| 对照组 | K01 | 008 | 2023-12-28 |  |  | 是 | Y | Y | Y |
| 对照组 | K01 | 058 | 2023-12-29 |  |  | 是 | Y | Y | Y |
| 对照组 | K01 | 059 | 2024-01-03 |  |  | 是 | Y | Y | Y |
| 对照组 | K01 | 112 | 2024-01-18 |  |  | 是 | Y | Y | Y |
| 对照组 | K01 | 114 | 2024-01-20 |  |  | 是 | Y | Y | Y |
| 对照组 | K01 | 139 | 2024-01-25 |  |  | 是 | Y | Y | Y |
| 对照组 | K01 | 140 | 2024-03-08 |  |  | 是 | Y | Y | Y |
| 对照组 | K01 | 177 | 2024-03-11 |  |  | 是 | Y | Y | Y |
| 对照组 | K01 | 180 | 2024-03-12 |  |  | 是 | Y | Y | Y |
| 对照组 | K02 | 017 | 2024-01-02 |  |  | 是 | Y | Y | Y |
| 对照组 | K02 | 019 | 2024-01-10 |  |  | 是 | Y | Y | Y |
| 对照组 | K02 | 023 | 2024-01-24 |  |  | 是 | Y | Y | Y |
| 对照组 | K02 | 024 | 2024-01-25 |  |  | 是 | Y | Y | Y |
| 对照组 | K02 | 026 | 2024-01-29 |  |  | 是 | Y | Y | Y |
| 对照组 | K02 | 028 | 2024-02-02 |  |  | 是 | Y | Y | Y |
| 对照组 | K03 | 037 | 2023-12-26 |  |  | 是 | Y | Y | Y |
| 对照组 | K05 | 047 | 2023-12-20 |  |  | 是 | Y | Y | Y |
| 对照组 | K05 | 049 | 2023-12-26 |  |  | 是 | Y | Y | Y |
| 对照组 | K05 | 051 | 2023-12-23 |  |  | 是 | Y | Y | Y |
| 对照组 | K05 | 053 | 2024-01-12 |  |  | 是 | Y | Y | Y |
| 对照组 | K05 | 055 | 2024-01-22 |  |  | 是 | Y | Y | Y |
| 对照组 | K05 | 093 | 2024-02-17 |  |  | 是 | Y | Y | Y |
| 对照组 | K05 | 094 | 2024-02-16 |  |  | 是 | Y | Y | Y |
| 对照组 | K06 | 083 | 2023-12-30 |  |  | 是 | Y | Y | Y |
| 对照组 | K06 | 084 | 2023-12-31 |  |  | 是 | Y | Y | Y |
| 对照组 | K06 | 085 | 2023-12-31 |  |  | 是 | Y | Y | Y |
| 对照组 | K06 | 086 | 2024-01-06 |  |  | 是 | Y | Y | Y |
| 对照组 | K06 | 089 | 2024-01-10 |  |  | 是 | Y | Y | Y |
| 对照组 | K06 | 091 | 2024-01-13 |  |  | 是 | Y | Y | Y |
| 对照组 | K06 | 149 | 2024-01-18 |  |  | 是 | Y | Y | Y |
| 对照组 | K06 | 151 | 2024-01-20 |  |  | 是 | Y | Y | Y |
| 对照组 | K06 | 153 | 2024-01-21 |  |  | 是 | Y | Y | Y |
| 对照组 | K06 | 156 | 2024-02-01 |  |  | 是 | Y | Y | Y |
| 对照组 | K06 | 204 | 2024-02-17 |  |  | 是 | Y | Y | Y |
| 对照组 | K06 | 207 | 2024-02-21 |  |  | 是 | Y | Y | Y |
| 对照组 | K06 | 208 | 2024-02-22 |  |  | 是 | Y | Y | Y |
| 对照组 | K06 | 227 | 2024-03-11 |  |  | 是 | Y | Y | Y |
| 对照组 | K06 | 228 | 2024-03-14 |  |  | 是 | Y | Y | Y |
| 对照组 | K07 | 063 | 2023-12-27 |  |  | 是 | Y | Y | Y |
| 对照组 | K07 | 064 | 2023-12-27 |  |  | 是 | Y | Y | Y |
| 对照组 | K07 | 065 | 2023-12-28 |  |  | 是 | Y | Y | Y |
| 对照组 | K07 | 068 | 2024-01-09 |  |  | 是 | Y | Y | Y |
| 对照组 | K07 | 069 | 2024-01-21 |  |  | 是 | Y | Y | Y |
| 对照组 | K07 | 070 | 2024-02-24 |  |  | 是 | Y | Y | Y |
| 对照组 | K09 | 121 | 2024-01-11 |  |  | 是 | Y | Y | Y |
| 对照组 | K09 | 124 | 2024-01-16 |  |  | 是 | Y | Y | Y |
| 对照组 | K09 | 125 | 2024-01-16 |  |  | 是 | Y | Y | Y |
| 对照组 | K09 | 128 | 2024-01-16 |  |  | 是 | Y | Y | Y |
| 对照组 | K09 | 158 | 2024-01-18 |  |  | 是 | Y | Y | Y |
| 对照组 | K09 | 160 | 2024-01-16 |  |  | 是 | Y | Y | Y |
| 对照组 | K09 | 161 | 2024-01-25 |  |  | 是 | Y | Y | Y |
| 对照组 | K10 | 115 | 2024-01-11 |  |  | 是 | Y | Y | Y |
| 对照组 | K10 | 118 | 2024-01-26 |  |  | 是 | Y | Y | Y |
| 对照组 | K10 | 120 | 2024-01-27 |  |  | 是 | Y | Y | Y |
| 对照组 | K10 | 190 | 2024-02-05 |  |  | 是 | Y | Y | Y |
| 对照组 | K10 | 192 | 2024-02-17 |  |  | 是 | Y | Y | Y |
| 对照组 | K10 | 193 | 2024-02-20 |  |  | 是 | Y | Y | Y |
| 对照组 | K10 | 194 | 2024-02-20 |  |  | 是 | Y | Y | Y |
| 对照组 | K10 | 198 | 2024-03-01 |  |  | 是 | Y | Y | Y |
| 对照组 | K10 | 200 | 2024-03-07 |  |  | 是 | Y | Y | Y |
| 对照组 | K10 | 201 | 2024-03-08 |  |  | 是 | Y | Y | Y |
| 对照组 | K13 | 142 | 2024-03-09 |  |  | 是 | Y | Y | Y |
| 对照组 | K13 | 148 | 2024-02-01 |  |  | 是 | Y | Y | Y |
| 对照组 | K14 | 183 | 2024-03-18 |  |  | 是 | Y | Y | Y |
| 对照组 | K14 | 184 | 2024-03-18 |  |  | 是 | Y | Y | Y |
| 对照组 | K14 | 186 | 2024-03-20 |  |  | 是 | Y | Y | Y |
| 对照组 | K14 | 236 | 2024-03-14 |  |  | 是 | Y | Y | Y |
| 对照组 | K14 | 237 | 2024-03-14 |  |  | 是 | Y | Y | Y |
| 对照组 | K14 | 239 | 2024-03-15 |  |  | 是 | Y | Y | Y |
| 对照组 | K15 | 009 | 2024-03-18 |  |  | 是 | Y | Y | Y |
| 对照组 | K15 | 012 | 2024-03-15 |  |  | 是 | Y | Y | Y |
| 对照组 | K15 | 014 | 2024-03-19 |  |  | 是 | Y | Y | Y |
| 对照组 | K15 | 220 | 2024-02-29 |  |  | 是 | Y | Y | Y |
| 对照组 | K15 | 222 | 2024-03-07 |  |  | 是 | Y | Y | Y |
| 对照组 | K15 | 224 | 2024-03-12 |  |  | 是 | Y | Y | Y |
| 对照组 | K16 | 211 | 2024-03-06 |  |  | 是 | Y | Y | Y |
| 对照组 | K16 | 212 | 2024-03-13 |  |  | 是 | Y | Y | Y |
