# Supplementary figures and images for: Efficacy and Safety of Lianhua Qingke Tablets in Children With Mycoplasma pneumoniae Pneumonia: A Randomized, Double‐Blind, Multicenter, Placebo‐Controlled Clinical Trial
Source: Clin Respir J. 2026 Jun 25;20(7):e70204. doi: 10.1111/crj.70204 (PMC13304230; doi:10.1111/crj.70204)

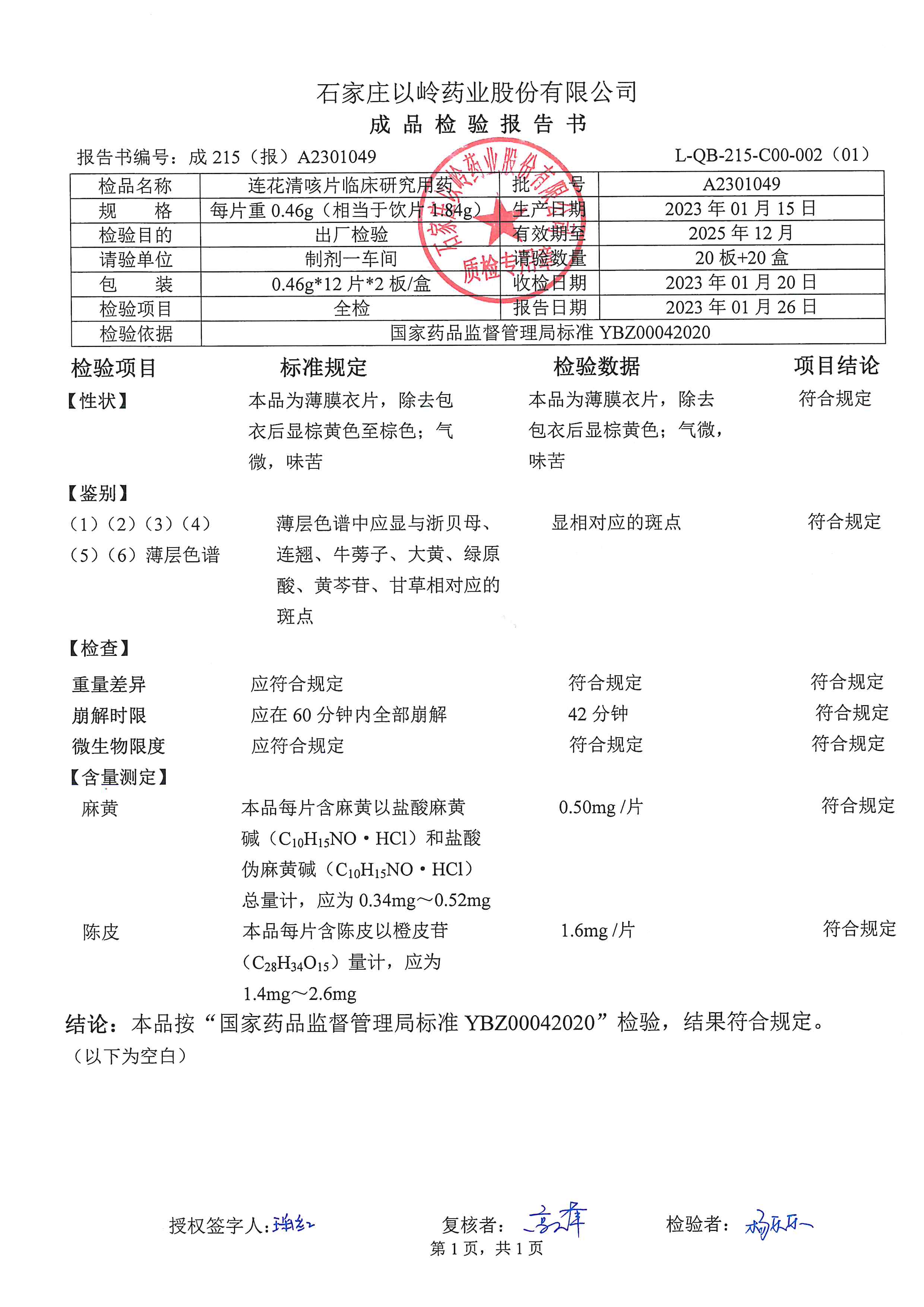

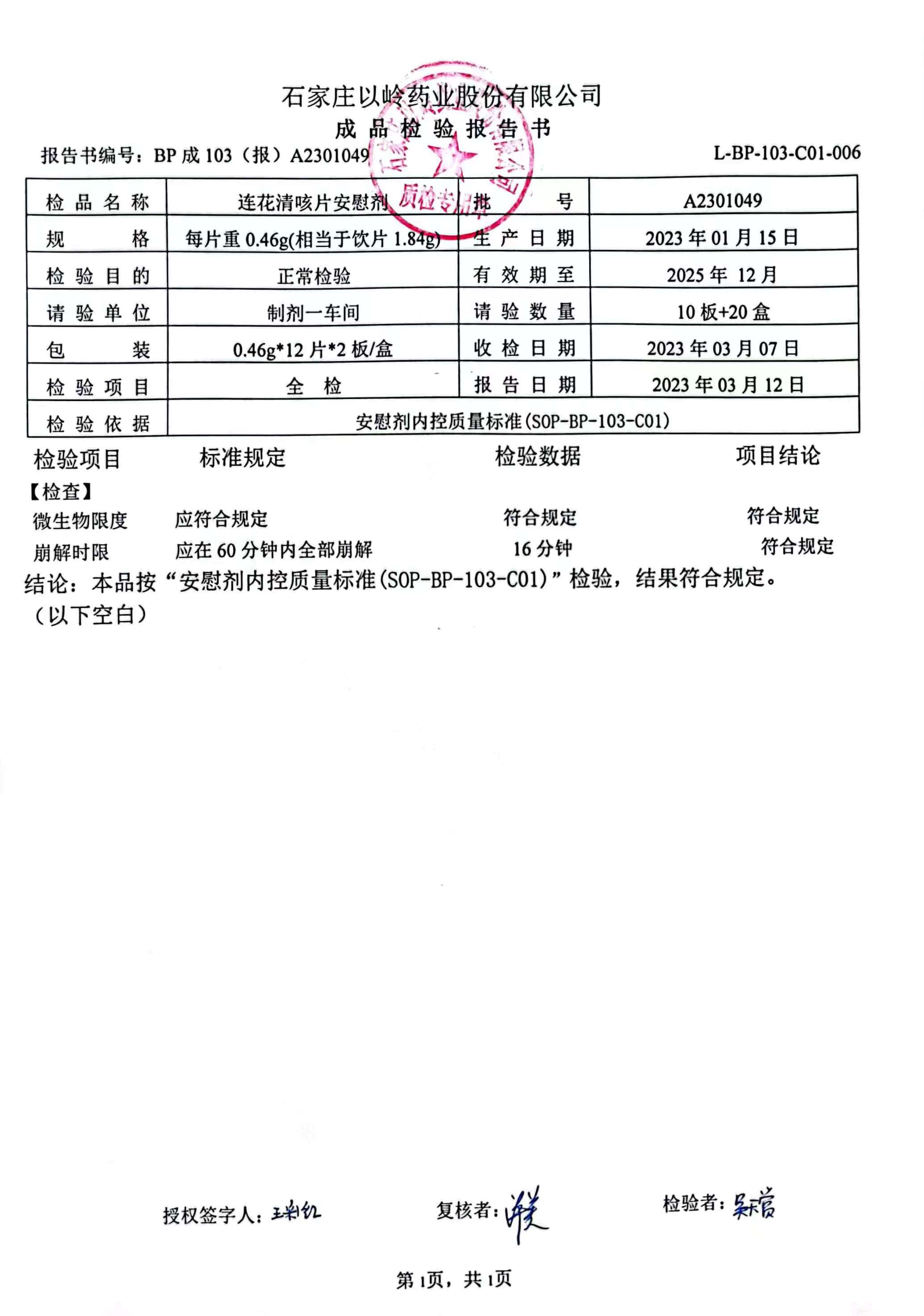

Supplement: Supplementary file 2 — Data S2: Supporting Information. [file CRJ-20-e70204-s001.docx]
