# Supplementary material for: Efficacy and Safety of Lianhua Qingke Tablets in Children With Mycoplasma pneumoniae Pneumonia: A Randomized, Double‐Blind, Multicenter, Placebo‐Controlled Clinical Trial
Source: Clin Respir J. 2026 Jun 25;20(7):e70204. doi: 10.1111/crj.70204 (PMC13304230; doi:10.1111/crj.70204)
Supplement: Supplementary file 4 — Table S1: Baseline characteristics of included (MPP, n = 128) vs. excluded (non‐MPP, n = 32) patients. [file CRJ-20-e70204-s004.docx]

**Supplementary Table S1. Baseline characteristics of included (MPP, n=128) vs. excluded (non-MPP, n=32) patients**

| Variable | Included (MPP, n=128) | Excluded (non-MPP, n=32) | P-value |
| --- | --- | --- | --- |
| Age (years, mean ± SD) | 7.3 ± 2.3 | 7.3 ± 2.4 | 0.876 |
| Male, n (%) | 71 (55.5) | 15 (46.9) | 0.376 |
| Illness duration (hours, median [IQR]) | 90.0 [70.0–102.0] | 89.0 [68.5–102.0] | 0.812 |
| Baseline CRP (mg/L, median [IQR]) | 7.38 [2.90–13.70] | 5.98 [2.58–12.55] | 0.545 |
| Baseline TCM syndrome total score (mean ± SD) | 16.9 ± 5.0 | 17.5 ± 5.0 | 0.542 |

Notes: Data for included patients (MPP) are from “raw data” SAR Table 2-1-1 (n=128). Data for excluded patients (non-MPP, n=32) were derived by subtracting MPP data from FAS data (raw data SAR Table 2-1, n=160). Continuous variables were compared using independent t-test or Wilcoxon rank-sum test as appropriate; categorical variables were compared using χ² test. No significant differences were observed between the two groups for any variable (all P > 0.05). Abbreviations: CRP, C-reactive protein; IQR, interquartile range; MPP, Mycoplasma pneumoniae pneumonia; SD, standard deviation; TCM, traditional Chinese medicine.
