# Supplementary material for: Efficacy and Safety of Lianhua Qingke Tablets in Children With Mycoplasma pneumoniae Pneumonia: A Randomized, Double‐Blind, Multicenter, Placebo‐Controlled Clinical Trial
Source: Clin Respir J. 2026 Jun 25;20(7):e70204. doi: 10.1111/crj.70204 (PMC13304230; doi:10.1111/crj.70204)
Supplement: Supplementary file 5 — Table S2: Detailed listing of treatment‐emergent adverse events (safety set). [file CRJ-20-e70204-s003.docx]

**Supplementary Table S2. Detailed listing of treatment-emergent adverse events (safety set)**

| Group | Adverse Event | n | Severity | Relationship to study drug | Outcome |
| --- | --- | --- | --- | --- | --- |
| LHQK (n=80) | Elevated red blood cell count | 1 | Mild | Unrelated | Resolved |
|  | Positive urine leukocytes | 1 | Mild | Unrelated | Resolved |
| Placebo (n=80) | Elevated platelet count | 4 | Mild | Unrelated | Resolved |
|  | Decreased serum creatinine | 2 | Mild | Unrelated | Resolved |
|  | Elevated alkaline phosphatase | 1 | Mild | Unrelated | Resolved |
|  | Decreased hemoglobin | 1 | Mild | Unrelated | Resolved |
|  | Decreased blood urea | 1 | Mild | Unrelated | Resolved |

*Note: No serious adverse events or adverse drug reactions occurred. All events were self-limited and required no medical intervention.*
